# Sphingosine 1‐phosphate lyase facilitates cancer progression through converting sphingolipids to glycerophospholipids

**DOI:** 10.1002/ctm2.1056

**Published:** 2022-09-20

**Authors:** Baasanjav Uranbileg, Makoto Kurano, Kuniyuki Kano, Eri Sakai, Junichi Arita, Kiyoshi Hasegawa, Takeshi Nishikawa, Soichiro Ishihara, Hiroharu Yamashita, Yasuyuki Seto, Hitoshi Ikeda, Junken Aoki, Yutaka Yatomi

**Affiliations:** ^1^ Department of Clinical Laboratory Medicine Graduate School of Medicine The University of Tokyo Tokyo Japan; ^2^ Department of Health Chemistry Graduate School of Pharmaceutical Sciences The University of Tokyo Tokyo Japan; ^3^ Hepato‐Biliary‐Pancreatic Surgery Division Department of Surgery The University of Tokyo Tokyo Japan; ^4^ Surgical Oncology and Vascular Surgery Division Department of Surgery The University of Tokyo Tokyo Japan; ^5^ Gastrointestinal Surgery Division Department of Surgery The University of Tokyo Tokyo Japan; ^6^ Division of Digestive Surgery Department of Surgery Nihon University School of Medicine Tokyo Japan

**Keywords:** glyceroLPLs (glycerolysophospholipids), GPR55 (G protein coupled receptor 55), LPG (lysophosphatidylglycerol), LPI (lysophosphatidylinositol), S1P (sphingosine 1‐phosphate), SPL (S1P lyase)

## Abstract

**Background:**

In addition to potent agonist properties for sphingosine 1‐phosphate (S1P) receptors, intracellularly, S1P is an intermediate in metabolic conversion pathway from sphingolipids to glycerolysophospholipids (glyceroLPLs). We hypothesized that this S1P metabolism and its products might possess some novel roles in the pathogenesis of cancer, where S1P lyase (SPL) is a key enzyme.

**Methods:**

The mRNA levels of sphingolipid‐related and other cancer‐related factors were measured in human hepatocellular carcinoma (HCC), colorectal cancer, and esophageal cancer patients’ tumours and in their adjacent non‐tumour tissues. Phospholipids (PL) and glyceroLPLs were measured by using liquid chromatography‐tandem mass spectrometry (LC‐MS/MS). In‐vitro experiments were performed in Colon 26 cell line with modulation of the SPL and GPR55 expressions. Xenograft model was used for determination of the cancer progression and for pharmacological influence.

**Results:**

Besides high SPL levels in human HCC and colon cancer, SPL levels were specifically and positively linked with levels of glyceroLPLs, including lysophosphatidylinositol (LPI). Overexpression of SPL in Colon 26 cells resulted in elevated levels of LPI and lysophosphatidylglycerol (LPG), which are agonists of GPR55. SPL overexpression‐enhanced cell proliferation was inhibited by GPR55 silencing. Conversely, inhibition of SPL led to the opposite outcome and reversed by adding LPI, LPG, and metabolites generated during S1P degradation, which is regulated by SPL. The xenograft model results suggested the contribution of SPL and glyceroLPLs to tumour progression depending on levels of SPL and GPR55. Moreover, the pharmacological inhibition of SPL prevented the progression of cancer. The underlying mechanisms for the SPL‐mediated cancer progression are the activation of p38 and mitochondrial function through the LPI, LPG‐GPR55 axis and the suppression of autophagy in a GPR55‐independent manner.

**Conclusion:**

A new metabolic pathway has been proposed here in HCC and colon cancer, SPL converts S1P to glyceroLPLs, mainly to LPI and LPG, and facilitates cancer development.

## Graphical abstract

- In human HCC, S1P lyase (SPL) levels were specifically and positively linked with levels of glycerophospholipids, including lysophosphatidylinositol (LPI).
- SPL facilitates cancer progression in both a GPR55 (LPI receptor)‐dependent and GPR55‐independent manner.
- The novel metabolic pathway, in which SPL converts sphingolipids to glycerophospholipids, might be a novel pharmacologic target for the cancer treatment.

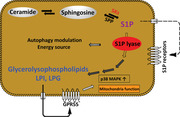

## 1 INTRODUCTION

Sphingosine 1‐phosphate (S1P) is one of the bioactive lipid mediators biosynthesized intracellularly from sphingosine by sphingosine kinases 1 and 2(SK1 and SK2). S1P regulates various cellular responses^1^, ^2^, ^3^, ^4^, ^5^, ^6^ through its specific G protein‐coupled receptors, S1P_1‐5_.^7^, ^8^, ^9^, ^10^ Among the various roles of S1P, its effects on cancer progression through its pro‐survival and anti‐apoptotic properties are highly significant. In the fields of oncology, the ceramide‐S1P rheostat theory has been proposed as being involved in the pathogenesis of cancer; according to this, up‐regulation of SKs is associated with conversion of ceramide, known as an anti‐survival mediator,^11^, ^12^, ^13^ into S1P, a pro‐survival mediator. In line with this ceramide‐S1P rheostat theory, up‐regulation of SKs has been demonstrated in various human cancers.^14^, ^15^, ^16^, ^17^, ^18^, ^19^ In our previous studies, however, we observed that in human hepatocellular carcinoma (HCC) and colon cancer tissues, as compared with the adjacent non‐tumour tissues, while the mRNA levels of the SKs were enhanced, the S1P levels remained low or unchanged, suggesting that up‐regulation of SKs does not always uphold the ceramide‐S1P rheostat theory.^20^, ^21^ Two distinct S1P‐degrading pathways are known to exist; one is dephosphorylation by S1P phosphatases (SPP),^22^, ^23^ and the other is irreversible cleavage by S1P lyase (SPL).^24^, ^25^ The low level of S1P in tumour tissues mentioned above may be explained as follows: SPL irreversibly degrades S1P at a greater rate than the rate of S1P generation by highly expressed SKs, since SPL, an S1P‐degrading enzyme, was also found to be up‐regulated in cancer tissues. Moreover, the ratio of SPL mRNA in HCC to that in non‐HCC tissues was higher in more undifferentiated HCC.^20^

SPL is responsible for sphingolipid degradation, as mentioned above, and the products also reportedly influence cell proliferation and survival by stimulating mitogenesis, independently from S1P.^26^ The endoplasmic reticulum, inner mitochondrial membrane, and cell membranes are the main sites of activity of SPL,^27^ and SPL is an integral membrane protein that is present in most tissues in mammals, being especially highly expressed in the intestine, liver, kidney and thymus; its expression is not known in platelets,^28^ but in white blood cells.^29^ SPL is reported to be down‐regulated in human colon cancer,^30^ but to be up‐regulated in ovarian cancer,^31^ and its expression level was especially increased in ovarian tumours that were resistant to chemotherapy.^32^ Despite the observations of altered expression levels of SPL in human tumour tissues, the roles of SPL in cancer pathogenesis have not yet been fully elucidated.

S1P was already reported in the 1960s to be metabolized into glyceroLPLs through the SPL pathway, and the roles of the enzymes involved in this pathway were also elucidated recently.^33^, ^34^ SPL converts S1P to hexadecenal (Hexa) and phosphoethanolamine (PE), and further, by the actions of the fatty aldehyde dehydrogenase (FALDH) and cytidine diphosphate (CDP)‐ethanolamine (CTP), the compound enters the glyceroLPLs synthetic pathway (Figure S1).^35^ GlyceroLPLs, including lysophosphatidic acid (LPA), lysophosphatidylcholine (LPC), lysophosphatidylethanolamine (LPE), lysophosphatidylglycerol (LPG), lysophosphatidylinositol (LPI) and lysophosphatidylserine (LPS) were studied widely as biologically active lipid mediators, and shown to have multiple roles in neurogenesis, vascular development, and immunity.^36^, ^37^ In addition to LPA, the role of which in carcinogenesis has been very well‐studied,^38^ involvement of the other glyceroLPLs in cancer has also been reported.^39^, ^40^, ^41^ We observed the possibility that minor glyceroLPLs might also have as important roles in cancer progression as S1P in human cancers, including HCC,^20^ colon cancer,^21^ and gastric cancer.^39^ In our previous studies overexpression of SPL enhanced cell proliferation, and silencing of SPL reduced cell proliferation in human HCC and colon cancer cell lines.^20^, ^21^

These previous results prompted us to hypothesize that S1P may not only be involved in the pathogenesis of cancer as a ligand for S1P receptors, but also as an intermediate metabolite in the metabolic conversion pathway from sphingolipids into glyceroLPLs. In this study, we demonstrate the importance of this metabolic pathway in the field of oncology.

## 2 MATERIALS AND METHODS

### 2.1 Materials

1‐Oleoyl (18:1) LPI, and (18:1) LPG (850100P, 858125P; Avanti Polar Lipids, Alabaster, AL) were dissolved in methanol. Just before use, the methanol was evaporated, and the reagents were resolved in PBS containing 1% fatty acid‐free BSA (A8806, Sigma‐Aldrich Co., St. Louis, MO). PE (16878, Cayman chemical, MI48108, USA) and Hexa (249084, Sigma‐Aldrich, Saint Louis, USA) were supplied as liquids and dissolved in PBS containing 1% fatty acid‐free BSA just before use.

CID (16020046; Cayman Chemical, Ann Arbor, MI, USA), Rapamycin (184‐02531, Fujifilm Wako Junyaku Co., Ltd., Osaka, Japan) and 2‐acetyl‐4‐tetrahydroxybutyl imidazole (THI) (T6330, Sigma‐Aldrich Co., St. Louis, MO) were dissolved in DMSO for the in‐vitro experiments, and in drinking water for the in‐vivo experiments.

### 2.2 Patients

We collected surgical samples from patients with HCC who had undergone liver resection (*N* = 148). The clinical characteristics of the subjects are shown in Table 1A. In addition, tumour samples from 26 colon cancer patients^21^ and samples obtained by endoscopy from the tumours and non‐tumour adjacent areas (tumour and non‐tumour tissues) of 33 esophageal cancer patients (Table 1B) at The University of Tokyo Hospital were also used for additional analyses. This study was conducted in accordance with the ethics guidelines of the 1975 Declaration of Helsinki, and with the approval of the Institutional Research Ethics Committee of the authors’ institution (Approval number 1143‐2). Written informed consent was obtained from the patients for the use of their clinical samples.

**TABLE 1:** Patients characteristics

A. HCC Patients	
Parameter	*n* = 148
Female/Male	27/121
Age (years)	70.5 (64.6–75.8)
Types of hepatitis	
Hepatitis B (%)	32 (21.6)
Hepatitis C (%)	49 (33.1)
Non B, non C (%)	66 (44.6)
Hepatitis B, C (%)	1 (.7)
Patients with primary HCC/recurrent HCC	95/53
Maximum tumour diameter (cm)	3.1 (2.0–5.5)
Number of tumours	
Single (%)	98 (66.2)
More than 2 (%)	50 (33.8)
White blood cell count (×10^3^/μl)	5.25 (4.40–6.37)
Hemoglobin content (g/dl)	13.5 (12.4–14.8)
Platelet count (×10^4^/μl)	15.8 (12.4–20.4)
C‐reactive protein CRP (mg/dl)	.07 (.03‐.20)
Albumin (g/dl)	4.0 (3.7–4.3)
Aspartate aminotransferase AST (U/L)	31.0 (24.0–52.7)
Alanine aminotransferase ALT (U/L)	29.0 (19.0–44.0)
Gamma‐glutamyl transferase GGT (U/L)	48.0 (29.0–104.7)
Total bilirubin (mg/dl)	.7 (.6–1.0)
Creatinine (mg/dl)	.81 (.68–.92)
Triglyceride (mg/dl)	99 (71.5–134.0)
Total cholesterol (mg/dl)	172 (150–194)
Fasting blood glucose (mg/dl)	103.5 (93.7–124.0)
HbA1c (NGSP) (%)	5.8 (5.5–6.6)
Prothrombin time‐ Int'l normalized ratio PT‐INR	.96 (.93–1.01)
ICGR15 (%)	12.0 (9.0–21.0)
Alpha‐fetoprotein AFP (ng/ml)	7.5 (3.9–44.9)
AFP‐L3 (%)	2.1 (.5–13.7)
Des‐gamma‐carboxy prothrombin DCP (mAu/ml)	37 (17.0–403.8)
Background liver	
Fibrosis stage 0 / 1 / 2 / 3 / 4	17/22/28/41/40
Activity grade 0 / 1 / 2	15/108/25
Tumour differentiation	
Well (%)	24 (16.2)
Well to moderate (%)	39 (26.4)
Moderate (%)	57 (38.5)
Moderate to poor (%)	21 (14.2)
Poor (%)	7 (4.7)
Microvascular invasion	(+)/(–) 36/112
Female/Male	3 / 30
Age (years)	71 (66–79)
Histology	
Squamous Cell Carcinoma/Adenocarcinoma	32/1
Cancer cell differentiation	
Well	4
Moderate	12
Poor	4
Undifferentiated	5
Tumour position	
Cervical	3
Upper thoracic	7
Middle thoracic	15
Lower thoracic	8
Lymph node metastasis (+/‐)	18/15
Recurrence	
Intra	3
Extra	12
White blood cell count (×10^3^/μl)	6.0 (4.9–8.0)
Hemoglobin content (g/dl)	13.8 (12.6–14.7)
Platelet count (×10^4^/μl)	23.4 (20.6–29.6)
CRP (mg/dl)	.10 (.05–.65)
Albumin (g/dl)	4.1 (3.9–4.3)
CYFRA (ng/ml)	2.3 (1.25–2.85)
SCC (ng/ml)	1.8 (1.1–2.5)
Anti‐p53 (U/ml)	.4 (.4–1.0)
Fibrinogen (mg/dl)	420 (379–528)
Brain natriuretic peptide BNP (mg/ml)	27.6 (18.6–44.3)
Tumour stage	
T I / II / III/ IV	7/3/17/6

Abbreviation: HCC, human hepatocellular carcinoma.

### 2.3 Cells and cell culture

The mouse colon cancer cell line, Colon 26, was obtained directly from the Cell Resource Center for Biomedical Research, Tohoku University (TKG 0518). Cells were maintained in RPMI‐1640(189‐02025, Fujifilm Wako Junyaku Co., Ltd., Osaka, Japan) containing 2000 mg/L glucose, 10% fetal bovine serum (FB‐1280/500, Biosera, Fujifilm Wako Junyaku Co., Ltd., Osaka, Japan), and 1% penicillin/streptomycin (168‐23191, Gibco, Grand Island, NY). The HCC cell line PLC/PRF/5 and colon cancer cell lines HCT116 and LoVo were directly obtained from RIKEN BioResource Center (Tsukuba, Ibaraki, Japan), and the HuH7 cells were directly obtained from the Health Science Research Resources Bank, Japan Health Science Foundation. The PLC/PRF/5 cells were maintained in RPMI‐1640, the HuH7 and HCT116 cells were cultured in DMEM, and the LoVo cells were cultured in Ham's F‐12(044‐29765, 087–08335 Fujifilm Wako Junyaku Co., Ltd., Osaka, Japan) supplemented with 10% fetal bovine serum.

### 2.4 Animal experiments

C57BL/6 mice purchased from CLEA Japan (Tokyo, Japan) were used in this study for evaluating tumour progression by intraperitoneal injection of the cancer cell lines. The mice were housed under a 12 h light/12 h dark cycle, with ad libitum access to food and water. All the animal experiments were conducted in accordance with the guidelines for the Care and Use of Laboratory Animals, and were approved by the Ethics Committee for Animal Experiments of the University of Tokyo (approval P17‐076). For the experiments using THI (SPL inhibitor), we used drinking water for administration of the chemical. To improve palatability, we added 5% dextrose into drinking water containing or not containing 25 mg/L of THI for the drug treatment and control groups, respectively (*N* = 10 mice/group).

### 2.5 Cell proliferation, migration and invasion assay

Cell proliferation in the colon cancer cell lines was analysed by measuring bromodeoxyuridine (BrdU) incorporation using the cell proliferation enzyme‐linked immunosorbent assay BrdU colorimetric assay kit (11647229001, Roche Applied Science, Upper Bavaria, Germany). Both serum‐ and glucose‐free culture medium was used to evaluate cell proliferation.

Cell migration and invasion assays were performed in accordance with the manufacturer's instructions (BD Biosciences, Franklin Lakes, NJ), as previously described.^20^

### 2.6 SPL overexpression or silencing

SPL stable transfection: Cells expressing human SPL protein were constructed using the mammalian cell expression vector p3FLAG CMV‐10 containing the corresponding cDNA derived from normal human liver RNA. The primers used for cloning were SPL: 5′‐ataagaatgcggccgctaaactatatgcctagcacaga‐3′ and 5′‐cggaattctcagtggggttttggagaaccattc ‐3′.

These primers were designed based on human SPL reference sequences (NM_003901). SPL cDNAs were generated by polymerase chain reaction and verified by DNA sequencing.

SPL stable silencing: We used the CRISPR/Cas9 system to inhibit the expression of SPL in Colon 26 cell lines, in accordance with the manufacturer's protocol (System Biosciences (SBI), CA, USA). The sgRNA‐encoding sequence targeting the *SPL* gene (5′‐tgcctctgcctgccaagtgc‐3′) was inserted into the CAS740A vector using two synthesized oligonucleotides (5′‐ atcctgcctctgcctgccaagtgc ‐3′ and 5′‐ aaacgcacttggcaggcagaggca ‐3′; FASMAC, Atsugi, Japan). Correctly inserted sgRNA‐encoding sequences were verified by sequencing using the Sanger method (FASMAC). To construct a donor vector for SPL, we used MCS1‐LoxP‐EF1a‐GFP‐T2A‐Puro‐P2A‐hsvTK‐pA‐LoxP‐MCS2(Cat#: HR110PA‐1, SBI). The left and right arms (∼800 bp each) were amplified from genomic DNA using primers (left arm: 5′‐ccggaattcatcttccagccagagagtaagtatactacc‐3′ and 5′‐ggaagatctagagctggcaagtgcccc‐3′; right arm: 5′‐cgcggatccaaaaaaaattatttaagcttatcaggaaga‐3′ and 5′‐acatgctacggagagtaactctgctgtaaagaggagc‐5′), and sequentially cloned into the vector by T4 DNA ligase (Toyobo, Osaka, Japan). All PCR products were verified by DNA sequencing (FASMAC).

For transient overexpression or knockdown of SPL in the liver and colon cancer cell lines, we followed a similar method as that described previously.^20^, ^21^

### 2.7 Transfection of the Crispr/Cas9, donor plasmid and selection

Colon 26 cells were transfected with Crispr/Cas9 and donor plasmid using Lipofectamine 3000(L3000‐008, Thermo Fisher Scientific, Massachusetts, USA), in accordance with the manufacturer's protocol. The following day, the cells were replaced with fresh medium, and re‐seeded when the cells became confluent. One week later, the transfected cells were subjected to selection with puromycin at a concentration of 5.0 μg/ml. Two weeks after the selection, the cell colonies were checked under fluorescence microscopy and sorted and expanded in 24‐well plates, followed by extraction of genomic DNA for genomic PCR using standard methods. To detect HR events, we performed junction PCR using primers derived from outside sequences and the donor vector sequence and then verified them by DNA sequencing of the junction PCR products.

### 2.8 Lysophospholipid, S1P and phospholipid measurements

Cell lysates were used (*N* = 6) for measurement of the lipid levels by LC‐MS/MS in SPL‐overexpressing or ‐silenced cells, and following addition of PE or Hexa. The lysophospholipid (LPL) and S1P measurements are described in previous reports.^42^, ^43^, ^44^, ^45^ In brief, for phospholipid (PL) measurement, the cell lysate samples were mixed and sonicated with methanol and an internal standard (.2 μM PC [37:4] or 2 μM PI, PE, PG, PS [37:4]). After centrifugation at 21,500 g, the resulting supernatant was transferred to a sample tube for LC–MS/MS analysis. Then, the methanol extract (20 μl) was analysed in an autosampler (Nanospace LC, Shiseido) equipped with a C18 CAPCELL PAK ACR column (1.5Å; 250 mm; Shiseido), and the PLs were extracted using a gradient of solvent A (20 mM ammonium formate in water) and solvent B (acetonitrile/iso‐propanol [50/50, v/v]). The eluate was sequentially ionized with electrospray ionization using a Quantum Ultra triple quadrupole mass spectrometer (Thermo Fisher Scientific). The concentrations of the S1P, PL and glyceroLPLs were calculated from the area ratio to the internal standard using calibration curves (Figure S2B–D).

### 2.9 Western blotting

Proteins from the cells were extracted with M‐PER Mammalian Protein Extraction Reagent (78501, Thermo Fisher Scientific) plus Protease Inhibitor cocktail (11873580001, Compete, Roche Molecular Diagnostics, CA, USA). The extracts were separated using sodium dodecyl sulfate‐polyacrylamide gel electrophoresis (SDS‐PAGE) and blotted on to Trans‐Blot, Turbo, Transfer Pack (Bio‐Rad) membrane, incubated with antibodies against Beclin1(1:1000 dilution) and LC3(1:1000 dilution), PD017 and PM036(MBL, Nagoya, Japan), total mTOR, p70S6K and phosphorylated mTOR (ser2448), phosphorylated p70S6K (Thr389), EGFR (1:1000 dilution each, 2983, 2708, 5536, 9234, and 4267; Cell Signaling Technology, Danvers, MA), β‐actin (1:2000 dilution), ABS528 (Merck Millipore), A5441(Sigma–Aldrich, St. Louis County, Missouri, USA), TFAM (1:1000 dilution; 19998‐1AP, Proteintech Group Inc, Rosemont, IL, USA) and PGC1(1:2000 dilution; ab54481, Abcam, Cambridge, UK). Immune‐reactive proteins were visualized using a chemiluminescence kit (RPN2232, Amersham ECL Prime, GE Healthcare, Chicago, USA) and recorded using the ChemiDoc imaging system (Bio‐Rad Laboratories, Inc. CA, USA). For quantification of the protein levels in on Western blotting (WB), the intensities of the bands were measured using the image analyser software, Image J (NIH ImageJ). WB bands from a total of 3 replicates of the same set of samples were used for the analyses.

### 2.10 Quantitative real‐time PCR for SPL

The total RNAs of the cell lines were extracted using the Gen Elute mammalian total RNA miniprep kit (1002787764, Sigma–Aldrich). One microgram of purified total RNA was transcribed using a SuperScript™ First‐Strand Synthesis System for RT‐PCR (04896866001, Roche Molecular Diagnostics, CA, USA). Quantitative real‐time PCR was performed with a TaqMan Universal Master Mix (4324018, Applied Biosystems by Life Technologies, Thermo Fisher Scientific) using the Verity Real Time PCR System (Applied Biosystems). SPL, GPR55 and internal control 18S ribosomal primers and probes (TaqMan Gene Expression Assays) were obtained from Applied Biosystems (Mm00486079, Mm02621622 and Hs99999901). The samples were incubated for 10 min at 95°C, followed by 40 cycles at 95°C for 15 s, 60°C for 1 min. The target gene mRNA expression level was quantified relative to ribosomal 18S using the 2−Ct method (Applied Biosystems, User Bulletin No 2).

### 2.11 Measurement of metabolites

Metabolomics analyses were performed with a gas chromatography–mass spectrometry (GC‐MS) system (QP2020; Shimadzu Corporation), as described previously.^46^, ^47^

### 2.12 Glucose uptake assay

We used the homogenous bioluminescent method for validation of the glucose uptake rate in different cell lines using the Glucose Uptake‐Glo Assay, Promega Corporation (J1342, Madison, USA), in accordance with the manufacturer's instructions. In brief, to detect 2‐deoxyglucose‐6‐phosphate (2DG6P), cells were cultured under normal conditions for 24 h, 2DG was added after medium removal, and incubation was continued for a further 10 min with further addition of the reaction buffers. Finally, 2DG6P detection reagent was added and after incubation for 30 min, the luminescences were recorded in the microplates containing different cell lines, using .3–1.0 integration on luminometer Synergy H1(BioTek, Vermont, USA).

### 2.13 Optical imaging of disseminated tumours in the peritoneal organs of the mice

2×10^6^ cells in 100 μl PBS were injected intraperitoneally on day 0, optical IRDye 2‐DG (LICOR IRDye 800CW 2‐DG, LICOR biosciences, Nebraska, USA) was injected into the tail vein of the mice on day 8, and on day 10, samples were collected for further analysis. Peritoneal organs (mostly small intestines, cecum) were used for the fluorescence imaging performed by the Odyssey CLx Imaging system. Identical illumination settings (channels, resolution, intensities, quality, analysis and focus) were used for the acquisition of all images. Images were acquired and analysed using Image Studio Ver. 4.0 software.

### 2.14 Mitochondrial membrane potential

Double‐fluorescence staining of mitochondria by JC‐1, either as green fluorescent J‐monomers or as red fluorescent J‐aggregates, was used for monitoring the mitochondrial membrane potential in the cell lines using the JC‐1 MitoMP Detection kit (MT‐09, Dojindo Co. Ltd., Kumamoto, Japan), in accordance with the manufacturer's instructions.

### 2.15 Statistical analysis

Data processing and analysis were performed using R statistical software version 3.3.1(http://www.r‐proje ct.org), and data were processed with the Graphpad Prism 8.0 software (GraphPad Software, San Diego, CA). For the clinical studies, the Wilcoxon signed‐rank test was used for the comparison between the tumour tissues and the corresponding non‐tumour tissues. Spearman rank correlation test was used to evaluate the correlations. For the basic experiments, unpaired t‐test or one‐way analysis of variance (ANOVA) were used to analyse differences in the mRNA levels of SPL, BrdU incorporation and levels of the LPL species, S1P levels and phospholipids in the cell lysates. The results of the lipid levels are expressed as the means and standard deviations (SD). The results were considered significant when the *p* values were <.05. All *p*‐values were described as true values except when p‐values were < .001.

## 3 RESULTS

### 3.1 Increased mRNA expression levels of SPL in the tumour tissues were positively correlated with the levels of several glyceroLPLs, especially LPC and LPI, in human HCC

**FIGURE 1:**
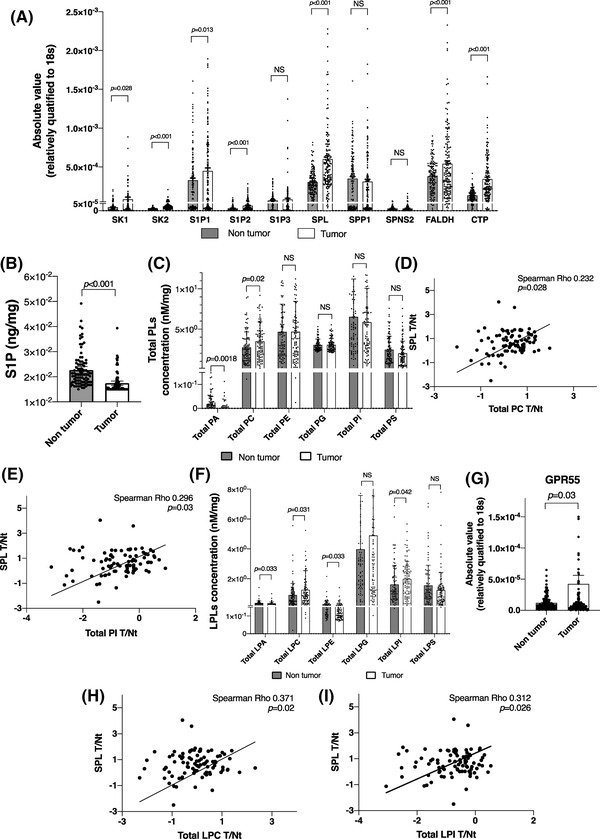
In human human hepatocellular carcinoma (HCC) tissue samples, among the sphingolipid‐related factors, the S1P lyase (SPL) mRNA expression levels were correlated with the total phospholipid and total lysophosphatidylcholine (LPC) and lysophosphatidylinositol (LPI) levels. (A) The mRNA expression levels of sphingolipid‐related factors in tumour specimens and specimens obtained from the adjacent non‐tumour tissues (hereinafter simply, tumour and non‐tumour tissues) in 148 patients with HCC. (B) S1P levels in the tumour and non‐tumour tissue specimens were measured by liquid chromatography‐tandem mass spectrometry (LC‐MS/MS) and corrected for the protein concentration. (C and F) Total PL and glycero‐LPL levels in human tumour and non‐tumour tissues. (D, E, H and I) A representative of a correlation between the SPL mRNA levels (tumour/non‐tumour ratio) and the PL (D and E) and glycero‐LPL levels (H and I). (G) GPR55 mRNA expression levels in the tumour and non‐tumour tissues. The differences between the tumour tissues and the corresponding non‐tumour tissues were statistically analysed by the Mann–Whitney *U* test. Spearman rank correlations were calculated to evaluate the correlations between the mRNA expression levels and absolute values of the PL, glycero‐LPL levels; *p* < .05 was considered as being indicative of statistical significance.

The mRNA expression levels of proteins involved in the metabolism of S1P, of cancer‐related factors, and levels of phospholipids (PL) and glyceroLPLs were measured in the tumour specimens and specimens of non‐tumour tissues (tumour and non‐tumour tissues) obtained from a total of 148 HCC patients. Similar to the findings in our previous study,^20^ we observed increased expression levels of SK1 and SK2 (Figure 1A), which are enzymes catalysing the generation of S1P, and clearly decreased levels of S1P (Figure 1B. The calibration curve used for the concentration calculation is shown in Figure S2B), in the tumour tissues as compared with the non‐tumour tissues. In regard to the level of SPL and SPP, the enzymes degrading S1P, the mRNA level of SPL, but not that of SPP, was also significantly higher in the tumour tissues. Expression levels of the downstream enzymes involved in glyceroLPLs production from sphingolipids, such as FALDH and CTP, were also higher in the tumour tissues. Among the S1P receptors, the expression levels of S1P_1_ and S1P_2_ were enhanced in the tumour tissues, while no significant changes were observed in the S1P_3_ expression levels (Figure 1A). Although considerable variation existed in several results maybe due to the difference in the process of handling specimens among the cases, since surgical specimens of tumour and non‐tumour were taken and processed together at the same time and compared with the paired tests, the difference shown in this clinical study could be reliable.

The results of the correlation analyses are shown in Table S1,2. Among the proteins involved in the metabolism and functioning of S1P, the mRNA level of SPL showed moderately positive correlations with the mRNA levels of FALDH and CTP (Table S1A), suggesting that all the enzymes involved in the irreversible degradation of S1P might be modulated in a similar manner. We classified the cancer‐related factors mainly into 5 groups by their functions, and among the factors that we examined, the strongest positive correlations of the S1P‐related factors were observed with markers of apoptosis and autophagy (Table S2A).

In regard to the phospholipid (PL) levels, the total PC levels were significantly higher and the total PA levels were lower in the tumour tissues (Figure 1C. The calibration curves used for concentration calculation are shown in Figure S2C), where the level of SPL (tumour/non‐tumour ratio) was positively correlated with the levels of PC, PG, PI and PS, as shown in Figure 1D,E and Table S3. Among the levels of the glyceroLPLs in the tumour tissues, the total LPC and LPI levels were higher, while the total LPA and LPE levels were significantly lower, with no changes in the total levels of LPG and LPS (Figure 1F. The calibration curves used for concentration calculation are shown in Figure S2D). The levels of LPC and LPI were significantly positively correlated with those of SPL (tumour/non‐tumour ratio), whereas no significant correlations of the levels of SPL with any other glyceroLPLs were observed (Figure 1H,I, Table S3). Interestingly, the mRNA levels of the S1P‐related factors (tumour/non‐tumour ratio) other than SPL (tumour/non‐tumour ratio) showed no significant correlations with the levels of the diacyl PLs or glyceroLPLs (tumour/non‐tumour ratio) (Table S3), suggesting that SPL might have crucial roles in the metabolic conversion pathway from sphingolipids to glyceroLPLs. Moreover, the mRNA level of GPR55, which is known as a receptor of LPI, was higher in the tumour tissues than in the non‐tumour tissues (Figure 1G). All these patient‐sample‐based results suggest the important role of SPL in cancer progression, as an enzyme promoting the generation of glyceroLPLs from sphingolipids.

### 3.2 The mRNA levels of SPL and their correlations with the levels of cancer‐related factors in colorectal cancer and esophageal cancer

As described in the *Introduction* section, based not only the findings in HCC, but also in human colorectal cancer, we hypothesized that SPL has a crucial role in cancer progression. Because of the limited amounts of tumour and non‐tumour tissues available from colon cancer patients, we could not measure the levels of lipids in these samples and could only investigate the correlations of SPL with cancer‐related factors, as described in Table S2B, and additional correlations among sphingolipid‐related factors (Table S1B). Similar to the findings in HCC, we observed quite strong positive correlations between the levels of SPL and autophagy markers. The mRNA expression levels of CTP, an enzyme that functions downstream of SPL, were significantly and strongly correlated with the expression levels of SPL, and moderately with the expression levels of FALDH, suggesting the involvement of the SPL pathway in colon cancer progression. In order to validate our results, we subsequently used The Cancer Genome Atlas (TCGA) database to analyse the correlations of SPL with sphingolipid‐related factors or various markers in the pan‐cancer study analysis (1210 cancer tissues). Most of the correlations were similar to our results (Table S1C, Table S2C).

Contrary to the modulation of the SPL expression levels in human HCC and colorectal cancer, we observed no significant modulation of the SPL levels in esophageal cancer, which showed only enhanced expression levels of the S1P_2_, S1P_3_ and S1P_5_ receptors (Figure S2A).

### 3.3 Modulation of the SPL expression levels led to not only changes in the cell proliferation, migration and invasion abilities but also in the levels of glyceroLPLs of the cancer cells

**FIGURE 2:**
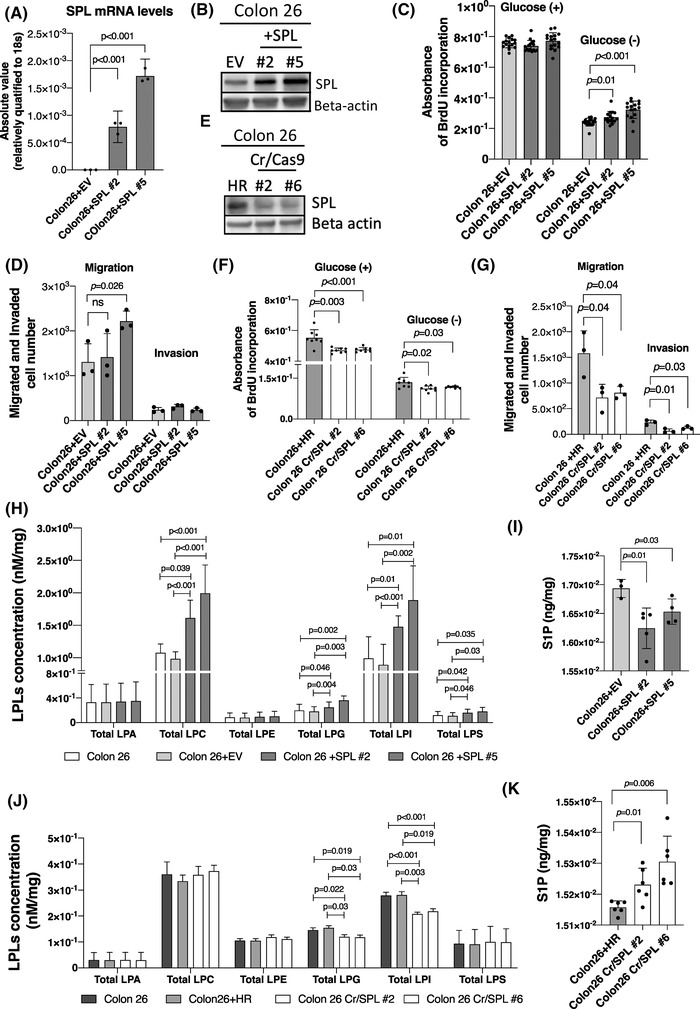
Modulation of the S1P lyase (SPL) expression level influences cell proliferation, migration, invasion and glyceroLPL levels mostly in glucose absent conditions. (A, B and E) Confirmation of stably overexpressed or inhibited SPL in the mouse colon cancer cell line Colon 26 by measuring the (A) mRNA expression levels (*N* = 3) and (B and E) protein levels. The mammalian cell expression vector p3FLAG CMV‐10 was used to induce stable overexpression, and the CRISPR/Cas9 system for stable inhibition. (C, D, F and G) The cell proliferation (*N* = 24), migration (*N* = 3) and invasion (*N* = 3) rates evaluated in the SPL‐overexpressing (C and D) and SPL‐inhibited cell lines (F and G), in the presence or absence of glucose. (H and J) The total glycero‐LPL levels in the SPL‐overexpressing and SPL‐inhibited cell lines. (I and K) S1P levels in cell lines in which the SPL expression levels were modulated. Measurement of the total glycero‐LPL and S1P levels (*N* = 6) were performed in the cell lysates and adjusted to the protein concentrations (which were used later for the analysis). Differences between the two groups were assessed using the unpaired Student's *t*‐test, while differences among more than two groups were assessed using a one‐way ANOVA, *p* < .05 was considered as being indicative of statistical significance. EV, empty vector transfected control cell line; HR, homologous recombination vector only transfected control cell line; +SPL, SPL overexpressing cell line; Cr/SPL, SPL knock out cell lines by using CRIPR/Cas9 system

We performed in vitro experiments in human HCC cell lines and human colon cancer cell lines (Figure S4) and the mouse colon cancer cell line, Colon 26. Since series of elegant studies suggested that S1P and glyceroLPLs are involved in tumour immune response,^48^, ^49^ in the present study, we aimed to investigate the roles of SPL in vivo in the presence of immune system, not with nude mice. Therefore, we mainly present the results obtained in the Colon 26 cell line rather than in human HCC or human colon cancer, for further applications in a murine xenograft model. To conduct a continuous in vitro study, first, we produced cell lines stably overexpressing SPL. The mRNA and protein expression levels of SPL in the cell lines are shown in Figure 2A,B (Figure S3O). We chose 2 cell lines, namely, Colon26+SPL#2 and Colon26+SPL#5, as one moderately overexpressing SPL and the other strongly overexpressing SPL (red circled colonies in Figure S3O). As depicted in Figure 2I, the levels of S1P in the two SPL‐overexpressing cell lines were significantly lower as compared with those in the empty vector‐transfected (Colon26+EV) control cell lines, confirming successful overexpression of functional SPL in the two experimental cell lines. The influence of SPL overexpression on cancer progression was examined by measuring the cell proliferation abilities of the cells; the cells were cultured for 24 h in a serum‐free condition, in the presence or absence of glucose in the culture medium. We performed the experiments in both glucose‐present and glucose‐absent conditions in a serum‐free medium, since the conditions of nutrition in the medium might affect the phenotypes of these cell lines, considering the possible roles of SPL in promoting the use of sphingolipids as fuels for the progression of cancer (Figure 2C). While no significant difference was observed in the glucose‐present condition, increased proliferation rates of the SPL‐overexpressing cells were observed in the glucose‐free condition as compared with that in the control cell lines (Colon 26 and Colon26+EV), consistent with the findings of our previous study, in which we used transient overexpression of SPL in human HCC and colon cancer cell lines.^20^, ^21^ Increased cell migration ability was observed only in the cell line strongly expressing SPL (Colon26+SPL#5), in the presence of glucose (Figure 2D). Cell invasion was not promoted in SPL overexpressing cell lines (Figure 2D) and the expression levels of the matrix metalloproteinase 2(MMP2), which mediates cell invasion, were not modulated, either (data not shown).

Next, we suppressed the expression of SPL using the Crispr/Cas9 system. Figure S3A shows representative cell colonies with the donor vector in comparison with the empty HR vector and the selected cell colonies by genotyping analysis, depicted in Figure S3B. Colon26+Cr/SPL#2 and Colon26+Cr/SPL #6 were selected for further analysis and the SPL protein expression levels were lower in these cell lines than in the control cells (Figure 2E). The S1P levels were also significantly higher in these cell lines (Figure 2K). Silencing of SPL inhibited the cell proliferation abilities of the cells, irrespective of the presence or absence of glucose in the culture medium (Figure 2F), and also reduced their cell migration and invasion abilities even in the presence of glucose (Figure 2G). The representative images of migrated or invaded cells in SPL overexpressed or inhibited cell lines in comparison to control cell lines are shown in Figure S3N.

Since we hypothesized that SPL is the key enzyme involved in the production of glyceroLPLs from sphingolipids, we investigated the modulations of the levels of glyceroLPLs in the cell lines. In the SPL‐overexpressing cell lines, we observed no significant changes in the levels of the glyceroLPLs in the glucose‐present condition, or indeed any changes in the cell proliferation abilities (Figure S3C). On the contrary, in the glucose‐absent condition, the total LPC, LPI, LPG, LPS and LPE levels, adjusted for the protein levels, were higher in the SPL‐overexpressing cells (Figure 2H). There were no changes in the total LPA levels (Figure 2H). The modulations of the molecular species of the glyceroLPLs are shown in Figure S3D–H. Concordantly, among the glyceroLPLs, the LPI and LPG levels were significantly increased in the SPL‐overexpressing HCC and colon cancer cell lines (Figure S4B,F). In addition, the levels of the glyceroLPLs were modulated in dependence on the SPL expression levels (Figure S3O,P)

In the SPL silenced Colon‐26 cells, the, total LPI and LPG levels were significantly lower, irrespective of the presence or absence of glucose in the culture medium (Figure 2J, Figure S3I). The modulations of the molecular species of the LPI and LPG are shown in Figure S3J–M. Silencing of SPL was clearly associated with a specific reduction in the levels of LPI and LPG among the glyceroLPLs, suggesting the crucial roles of LPI and LPG in the metabolic conversion pathway from sphingolipids to glyceroLPLs, which is mediated by SPL. In the HuH7 and HCT116 cells treated with siRNA against SPL, the cellular LPG levels were also significantly lower (Figure S4D,H).

### 3.4 Rescue of the inhibited cell proliferation in SPL‐silenced cells by addition of LPI, LPG, PE and/or Hexa

**FIGURE 3:**
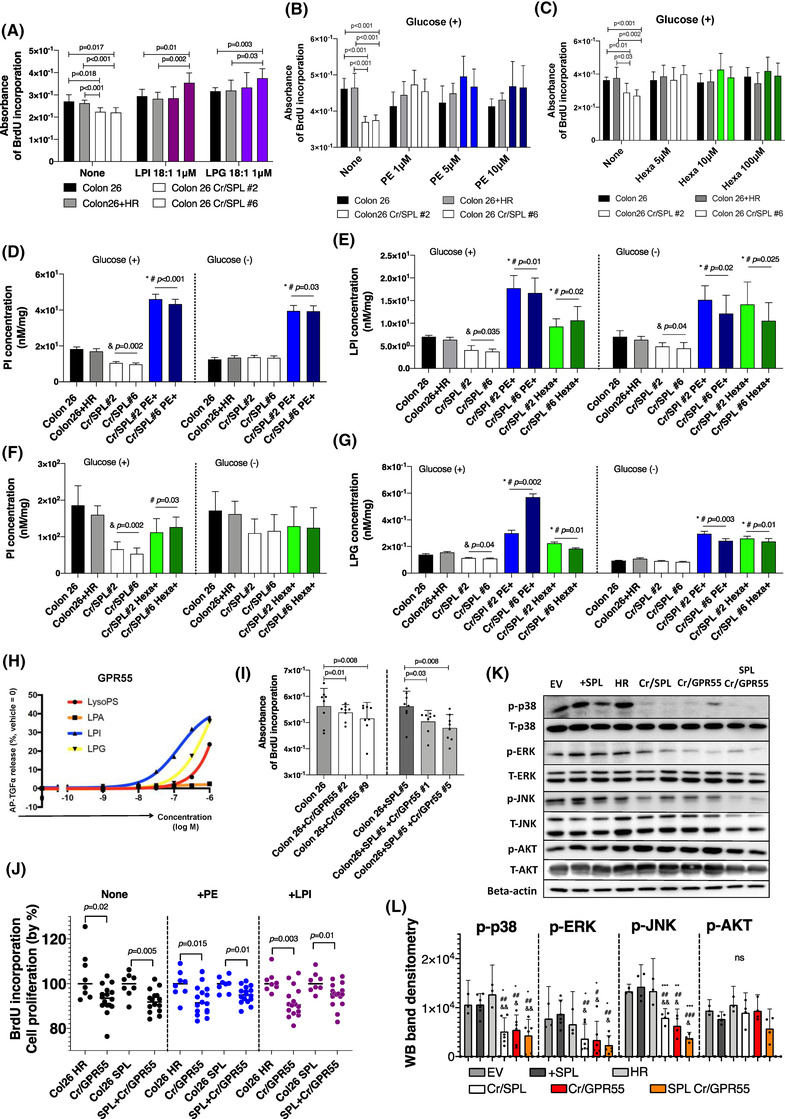
Addition of not only lysophosphatidylinositol (LPI) and lysophosphatidylglycerol (LPG), but also intermediate products of the S1P lyase (SPL)‐mediated metabolic pathway, reversed the retarded cell proliferation in SPL‐inhibited cell lines. (A) Analysis of cell proliferation in SPL‐inhibited cell lines further with the addition of LPI 18:1 or LPG 18:1 at a concentration of 1 μM for 24 h of incubation. Black and gray colour‐filled bars show control cell lines, and white bars show two selected colonies of SPL‐inhibited cell lines, with cherry red indicating the addition of LPI and purple indicating the addition of LPG (*N* = 8). (B and C) Cell proliferation analysis performed (*N* = 8) in the SPL‐inhibited cell lines by adding PE (B) and Hexa (C) at three different concentrations under glucose present conditions. (D and F) PI levels were measured by liquid chromatography‐tandem mass spectrometry (LC‐MS/MS) in SPL‐inhibited and control cell lines with or without the addition of PE (5 μM; blue) and Hexa (10 μM; green). (&) indicates the difference between the control and SPL‐inhibited cell lines, (*) indicates the difference between the control, and SPL‐inhibited and PE‐ or Hexa‐added cell lines, (#) indicates the difference between the control, and SPL‐inhibited and PE‐ or Hexa‐added cell lines; *p* < .05. (E and G) The same cells in Figure 3D and 3F were used for the measurements of the total LPI and LPG. (&) indicates the difference between the control and SPL‐inhibited cell lines, (*) indicates the difference between the control, and SPL‐inhibited and PE‐ or Hexa‐ added cell lines, (#) indicates the difference between SPL inhibited cell lines without PE/Hexa addition, and SPL‐inhibited and PE‐ or Hexa‐ added cell lines. (H) alkaline phosphatase transforming growth factor‐α (AP‐TGFα) release responses of GPR55 to glycero‐LPLs. A strong response was observed for LPI and LPG. (I) Cell proliferation assay performed in GPR55‐receptor‐inhibited cell lines; left: Colon 26 cell lines; right: SPL‐overexpressing cell lines. Two single‐colony cell lines were used for two different sets of experiments. Gray and dark gray bars represent control cell lines without GPR55 inhibition, white and light gray bars represent GPR55‐inhibited cell lines (*N* = 8). (J) Cell proliferation assays performed in three different cell lines (*N* = 16) and control cell lines (*N* = 8) when PE (5 μM, blue dots) and LPI 18:1 (1 μM, cherry red dots) were added. (K) Western blotting (WB) analysis of total and phosphorylated AKT, JNK, ERK, p38, and beta‐actin in cell lines with different expression levels of SPL and GPR55, including control cell lysates. (L) Quantification of WB by densitometry using the NIH ImageJ. WB bands from three repeats of the same set of samples were used, and the phosphorylation rates were quantified. (*) indicates differences with EV, (#) indicates differences with SPL overexpressing cell line, (&) indicates differences with HR. (*), (#), (&): *p* < .05; (**), (##), (&&): *p* < .01; (***), (###), (&&&): *p* < .001. Differences between the two groups were statistically analysed using the unpaired Student's *t*‐test, and differences more than two groups were analysed using one‐way ANOVA. EV, empty vector transfected control cell line; HR, homologous recombination vector only transfected control cell line; +SPL, SPL overexpressing cell line; Cr/SPL, SPL knock out cell lines by using CRIPR/Cas9 system; Cr/SPL+PE or Hexa, phosphoethanolamine (PE) or hexadecenal (Hexa) added in culture medium of the SPL knock out cell lines by using CRIPR/Cas9 system; Cr/GPR55, GPR55 knock out cell lines by using CRIPR/Cas9 system; SPL+Cr/GPR55, GPR55 knock out cell lines by using CRIPR/Cas9 system in SPL overexpressing cell line

Next, to clarify the involvement of LPI and LPG in the pathway mediated by SPL, we investigated whether the inhibited cell proliferation in SPL‐silenced cell lines would be rescued by the addition of LPI 18:1 or LPG 18:1 at a concentration of 1 μM each. Addition of LPI/ LPG not only reversed (Colon26 Cr/SPL#2), but rather enhanced (Colon26 Cr/SPL#6) the cell proliferation abilities of the SPL‐silenced cell lines (Figure 3A).

When SPL irreversibly cleaves S1P, as mentioned in the *Introduction* section, Hexa and PE are formed as intermediate products.^24^, ^26^ We further investigated whether addition of these two intermediate products might rescue the inhibited cell proliferation abilities and reduced production of glyceroLPLs in the SPL‐silenced cell lines. As shown in Figure 3B, addition of PE at a concentration of 1 μM, 5 μM, or 10 μM rescued the inhibited cell proliferation abilities to levels comparable to those of the control Colon 26 and Colon 26+HR cell lines. Similar results were obtained when we added Hexa (5 μM, 10 μM, or 100 μM) to rescue the proliferation ability of the SPL‐silenced cells (Figure 3C). The levels of phosphatidylinositol (PI) were increased in the SPL‐silenced cell lines added with PE or Hexa as compared with the control or SPL inhibited cell lines (Figure 3D,F). In regard to the levels of the glyceroLPLs, we observed increased levels of LPI and LPG in the SPL‐silenced cell lines added with PE or Hexa, as shown in Figure 3E,G. The effects on the levels of other PLs and glyceroLPLs are shown in Figure S5C–K. These results suggest that not only the final products of S1P metabolism (glyceroLPLs), but also intermediate products can rescue the inhibited cell proliferation ability of cancer cells induced by the silencing of SPL, suggesting that SPL plays a crucial role in cancer progression by catalysing the metabolic conversion pathway from sphingolipids to glyceroLPLs.

### 3.5 The LPI, LPG‐GPR55 axis is involved in the enhanced cell proliferation abilities of SPL‐overexpressing cells

LPI is an established agonist of GPR55, and LPG has also been reported to induce cellular responses through its specific receptor GPR55.^50^, ^51^, ^52^ Actually, a transforming growth factor alpha (TGFα) shedding assay showed the response of the GPR55 with LPI and LPG, as shown in Figure 3H. There are several reports indicating that the signaling pathways activated by LPI via its receptor GPR55 play crucial roles in different cancers.^53^, ^54^, ^55^, ^56^ Therefore, we next investigated the effects of inhibition of the GPR55 receptor by silencing its expression using the Crispr/Cas9 system, or using a GPR55 receptor antagonist (CID) at different concentrations in cell lines overexpressing and not overexpressing SPL. The GPR55 expression levels in the Colon 26 cell lines before silencing were similar to those in tissue samples of the mouse spleen, which is known to express GPR55 at high concentrations (Figure S5A). In the GPR55‐silenced cell lines, independently of the overexpression of SPL, the cell proliferation abilities were significantly retarded (Figure 3I). Concordant with the results obtained with genetic inhibition, pharmacological inhibition with CID at high concentrations also inhibited the cell proliferation abilities of the cell lines, regardless of whether the cells overexpressed SPL or not (Figure S5B).

We also used intermediate products from S1P degradation, such as PE, Hexa and the final products of this pathway, such as LPI and LPG, to confirm the role of GPR55 in the enhanced cell proliferation ability induced by the SPL‐mediated metabolic pathway. The cell proliferation abilities were not reversed by the addition of PE, Hexa, LPI, or LPG (partial in this case) in the cells lines with either genetically or pharmacologically inhibited GPR55 as compared with the control cell lines (Figure 3J). Based on the previous reports demonstrating that LPI inducing cell migration and cell proliferation,^40^ and that LPI also promotes inflammation by inducing phosphorylation of p38,^57^ we performed WB analysis to check the involvement of several possible cascades in cell lines with modulated SPL expressions or GPR55 silencing, as shown in Figure 3K. The results clearly indicate full inactivation of p38, and partial inactivation of ERK and JNK in the SPL‐modulated or GPR55‐silenced cell lines. The band intensities of p‐p38, p‐ERK, p‐JNK and p‐AKT on WB were quantified, as shown in Figure 3L. There were no differences in p‐p38 between control (EV) and SPL overexpressing cell lines (+SPL) in the glucose‐present conditions. However, when we cultured cells for 24 h in glucose‐free conditions, the phosphorylation rates of p38 in SPL overexpressing cell were significantly higher than those in control cells (Figure S5P,Q). These results demonstrate the importance of LPI and LPG, which are products of degradation of sphingolipids, and their induction of cancer cell proliferation via activation of p38 through the specific receptor GPR55.

All of these results, together with previous reports, clearly confirm our hypothesis that SPL is closely involved in cancer progression through acting as a key enzyme in the metabolic conversion pathway from sphingolipids to glyceroLPLs.

### 3.6 Peritoneal cancer dissemination was dependent on both the SPL expression levels and GPR55 expression levels

Next, we investigated whether modulation of the SPL and GPR55 expression levels might affect the cell proliferation activity in xenograft models in vivo. To quantify the dissemination of peritoneal cancer, we used 2‐deoxyglucose (2‐DG) probes. As described in Figure S6A, we first confirmed that the glucose uptake rates of the all the cell lines used for injection were similar. In Figure S6B, we have illustrated our in vivo experiment schedule. Briefly, we injected the cells (2×10^6^ cells in 100 μl PBS) intraperitoneally on day 0, injected IRDye 2‐DG into the tail vein of the mice on day 8, and on day 10, we collected samples for further analysis.

**FIGURE 4:**
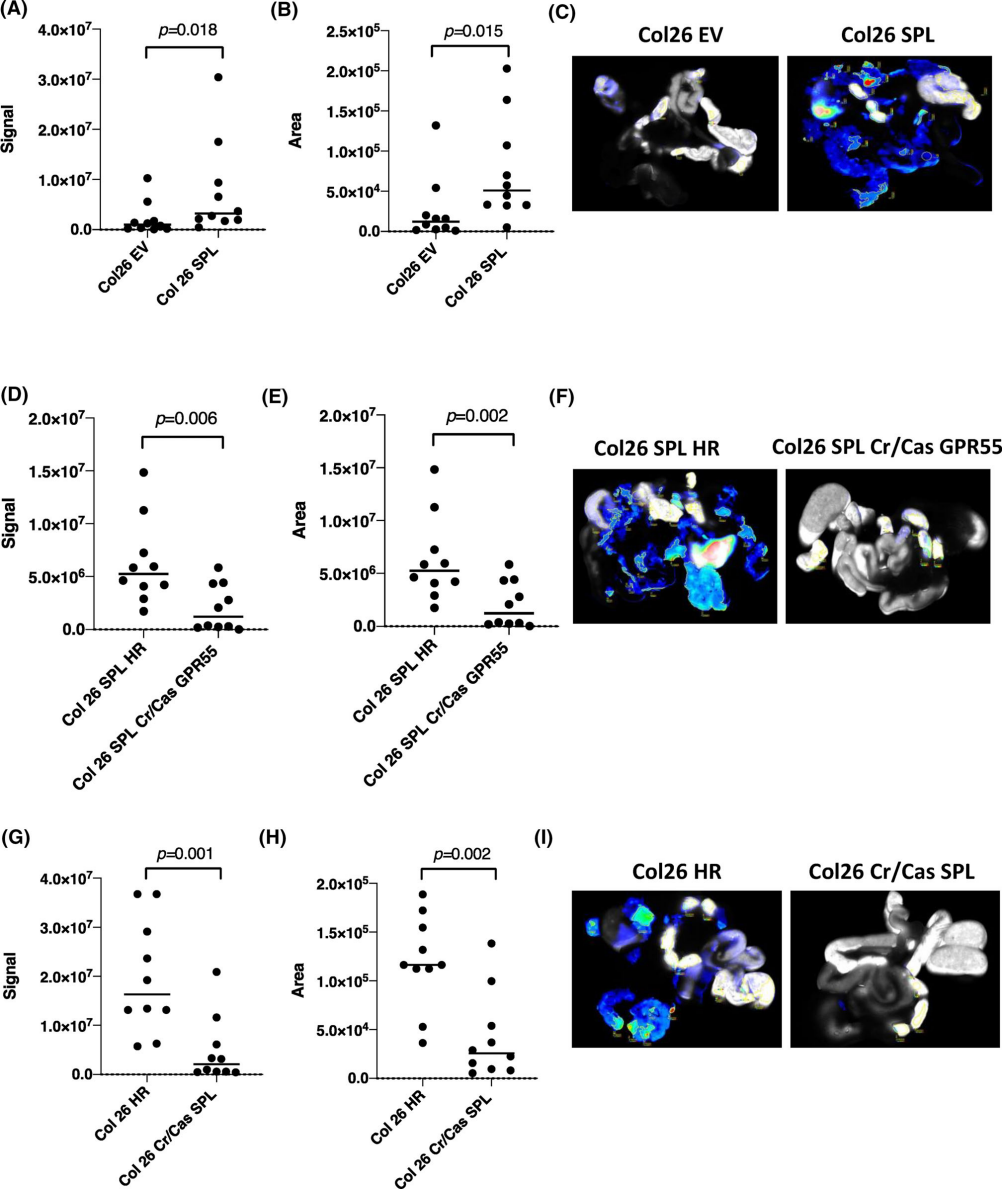
Effect of modulation of the S1P lyase (SPL) expression level on peritoneal cancer dissemination. Signal (A, D and G) and area (B, E and H) of the disseminated peritoneal cancer were analysed using the Odyssey CLx Imaging system. Identical illumination settings (channels: 700/800, resolution: 42 μM, intensities: auto, quality: lowest, analysis: small animal, and focus: 2.0 mm) were used for acquiring all images. (C, F and I) Representative scanned images from each group. Images were acquired and analysed using Image Studio Ver. 4.0 software. Differences between the two groups were statistically analysed by the unpaired Student's *t*‐test. EV, empty vector transfected control cell line; HR, homologous recombination vector only transfected control cell line; SPL, SPL overexpressing cell line; SPL HR, homologous recombination vector transfected SPL overexpressing cell line; Cr/Cas SPL, SPL knock out cell lines by using CRIPR/Cas9 system; SPL Cr/Cas GPR55, GPR55 knock out cell lines by using CRIPR/Cas9 system in SPL overexpressing cell line

We measured the 2‐DG signals (Figure 4A,D,G) and areas (Figure 4B,E,H) of the disseminated peritoneal cancer under the same conditions in all the samples. Figure 4C,F,I shows representative scanned images of the peritoneal organs with disseminated cancer (scanned images of the all other samples [*N* = 10] are shown in Figure S6C–E). HE stained histopathology images and original photos of mainly intestine and liver tissues with disseminated peritoneal cancer are shown in Figure S7A‐C. Significant differences were observed between the SPL‐overexpressing cell lines and SPL‐overexpressing, but GPR55‐silenced cell lines, as compared with their control cell lines. Overexpression of SPL was associated with a high dissemination rate of the cancer cells, which was, however, inhibited by additional silencing of GPR55 in this cells (Figure 4A,B,4D,E). In contrast, silencing of SPL was associated with very low 2‐DG signal and area as compared with that observed in the tissue samples obtained from the control cell line‐injected mice (Figure 4G,H). By these in vivo experiments, we could prove and reproduce the findings of our in vitro experiments performed using genetically modified SPL cell lines.

### 3.7 SPL modulates autophagy by providing the necessary energy to the cancer cells and maintains the mitochondrial membrane potential

**FIGURE 5:**
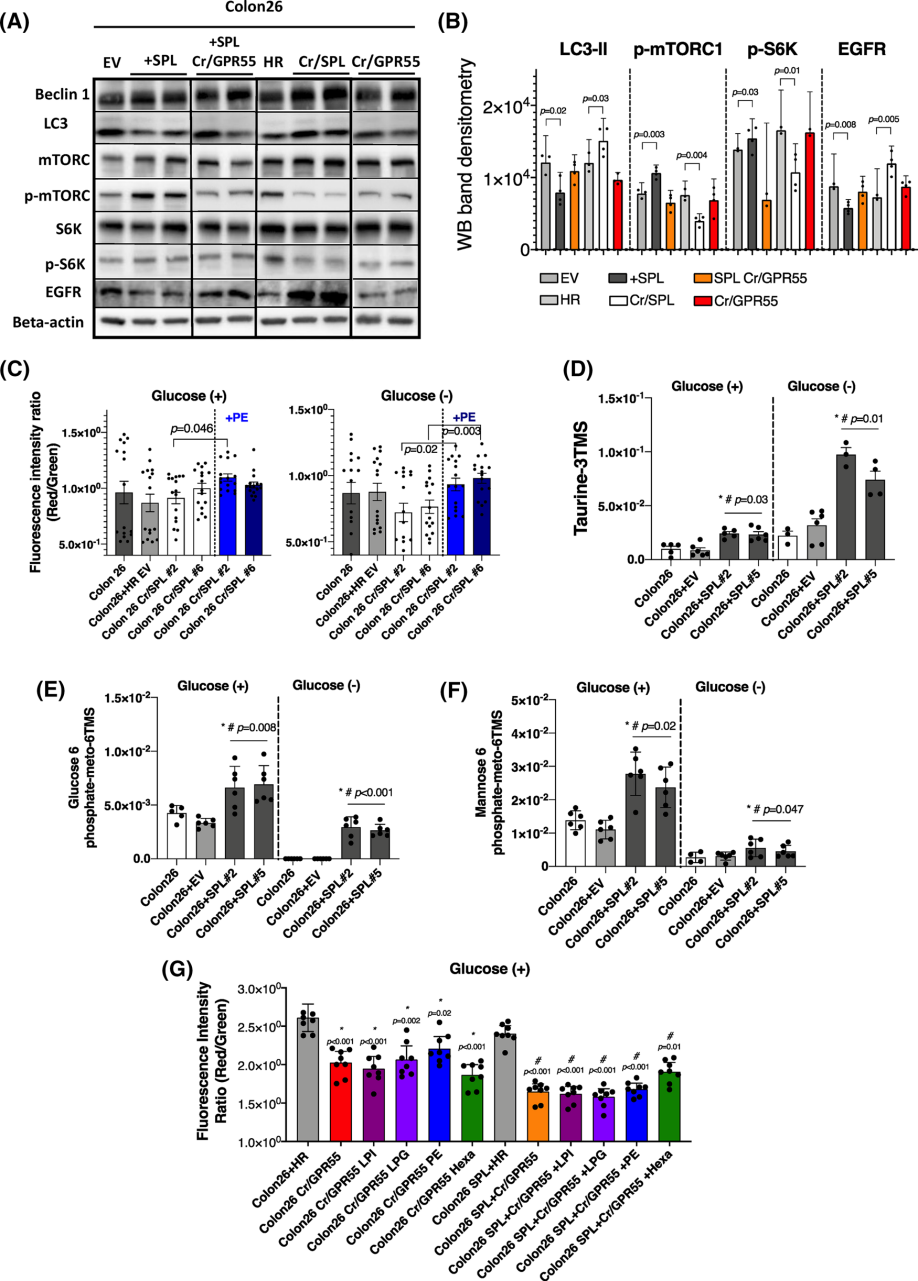
S1P lyase (SPL) modulates autophagy independently from GPR55 and maintains the mitochondrial membrane potential. (A) Western blotting (WB) analysis of the proteins involved in autophagy in cell lines with different expression levels of SPL or GPR55 as compared to control cell lines. (B) WB quantification by densitometry using the NIH ImageJ software. The bands from a total of three replicates of the same set of samples were used, and the protein levels or the phosphorylation rates were quantified. (C) Mitochondrial membrane potential was measured in SPL‐inhibited cell lines with or without addition of PE (5 μM) in the presence or absence of glucose. (D, E and F) The metabolites (taurine, glucose 6‐phosphate, mannose 6‐phosphate) were measured using GC‐MS in SPL‐overexpressing cell lines, in which levels were increased compared to control cell lines. (*) indicates the difference between the control and SPL‐overexpressing cell lines, (#) indicates the difference between the EV‐transfected control and SPL‐overexpressing cell lines. (G) Mitochondrial membrane potential was measured in GPR55‐inhibited cell lines with or without SPL overexpression by adding lysophosphatidylinositol (LPI) 18:1 (1 μM), lysophosphatidylglycerol (LPG) 18:1 (1 μM), PE (5 μM) and Hexa (10 μM). There were no changes with the addition of any of the compounds in glucose‐present condition. (*) indicates differences with HR control cell line, (#) indicates differences with SPL overexpressing HR control cell line. Differences between the two groups were statistically analysed by the unpaired Student's t‐test, while differences among more than two groups were statistically analysed by one‐way ANOVA. EV, empty vector transfected control cell line; HR, homologous recombination vector only transfected control cell line; SPL, SPL overexpressing cell line; SPL HR, homologous recombination vector transfected SPL overexpressing cell line; Cr/Cas SPL, SPL knock out cell lines by using CRIPR/Cas9 system; Cr/GPR55, GPR55 knock out cell lines by using CRIPR/Cas9 system in SPL overexpressing cell line; Cr/GPR55 LPI, or LPG, or PE, or He, GPR55 knock out cell lines by using CRIPR/Cas9 system with addition of the LPI, or LPG, or PE, or He. SPL Cr/Cas GPR55: GPR55 knock out cell lines by using CRIPR/Cas9 system in SPL overexpressing cell line; SPL Cr/Cas GPR55 + LPI, or LPG, or PE, or He, GPR55 knock out cell lines by using CRIPR/Cas9 system in SPL overexpressing cell line with addition of the LPI, or LPG, or PE, or He

As we observed positive correlations between S1P‐related factors and autophagy markers in the patients’ samples (Table S2), we investigated the protein expression levels of autophagy‐related factors in SPL‐overexpressing and SPL‐silenced cell lines. We observed down‐regulation of autophagy in the SPL‐overexpressing cell lines (Figure 5A). The expression of LC3‐II, which is known to play a crucial role in the formation of autophagosome, was clearly decreased. Also, as an inhibitor of autophagy, mTORC1 was activated in SPL‐overexpressing cells by phosphorylation at Ser2448. p70S6K was also phosphorylated by dual signaling, that is, by direct activation by activated mTORC1, and by interruption of inhibition of the autophagy pathway. The intensity of the bands corresponding to LC3‐II, p‐mTORC1, p‐S6K and ERK on WB were quantified as described in Figure 5B. Similar results were obtained from the peritoneal disseminated cancer tissue samples (Figure S6H), where we injected the cell lines of which the SPL and/or GPR55 expression levels were modulated.

In SPL‐silenced cell lines, we observed that the silencing of SPL enhanced autophagy. To demonstrate the roles of autophagy in cancer progression, we performed metabolomics analyses, using GC‐MS. In SPL‐overexpressing cell lines, the levels of several metabolites that serve as energy sources and play important roles in cell membrane stabilization and protein traffic were increased (Figure 5D–F). In addition, epidermal growth factor receptor (EGFR) protein expression levels were also different in the cell lines in which the expression levels of SPL were modulated (Figure 5A,B); EGFR is known to not only be linked to extracellular signals to control cell survival, growth, proliferation and differentiation,^58^ but also to determine the faith of autophagy in cancer.^59^

Table S2 also highlights the association between SPL and mitochondrial function. Actually, the mitochondrial membrane potential, which indicates cell health, was increased by the addition of PE in SPL‐silenced cell lines (Figure 5C), especially in the glucose‐absent condition. The representative fluorescence images of the mitochondrial membrane potential of these cells are shown in Figure S5L. When we treated the cells with Rapamycin, an inducer of autophagy, we did not observe the increasing effects of SPL overexpression on the cell proliferation and mitochondrial membrane potential, suggesting that the modulation of autophagy by SPL might be involved in the cancer‐progressing properties of SPL in the present experiments (Figure S5N,O).

Regarding mitochondrial function, the protein expression levels of the TFAM and PGC1, a mitochondrial transcription factor and a transcriptional coactivator, respectively, are shown in Figure S6H. Concordant with the results of the mitochondrial membrane potential in vitro, these two protein expression levels were enhanced in the cancer tissues of the mice injected with SPL overexpressing cells, but declined in those of the mice injected with SPL or GPR55 deleted cells.

Autophagy was not modulated in the cell lines in which the GPR55 receptor was inhibited, independent from SPL expression levels (Figure 5A). Mitochondrial membrane potential was decreased in the GPR55‐inhibited cell lines, which also showed a shift towards low cell proliferation rates, while the mitochondrial membrane potential could not be recovered by the addition of LPI, LPG, or PE or Hexa in the GPR55‐inhibited cell lines, in either the glucose‐present (Figure 5G) or glucose‐absent (Figure S5M) condition. These results suggest that the modulation of autophagy by SPL might not be associated with the SPL‐LPI, LPG‐GPR55 pathway, whereas that of the mitochondrial function might be mediated by this axis.

These results suggest that high levels of SPL could maintain the necessary energy and nutrition of the cells by inducing metabolites and modulating autophagy in cancer cell lines.

### 3.8 The SPL‐mediated pathway in which sphingolipids are converted into glyceroLPLs might be a potential pharmacological target for the treatment of cancer

**FIGURE 6:**
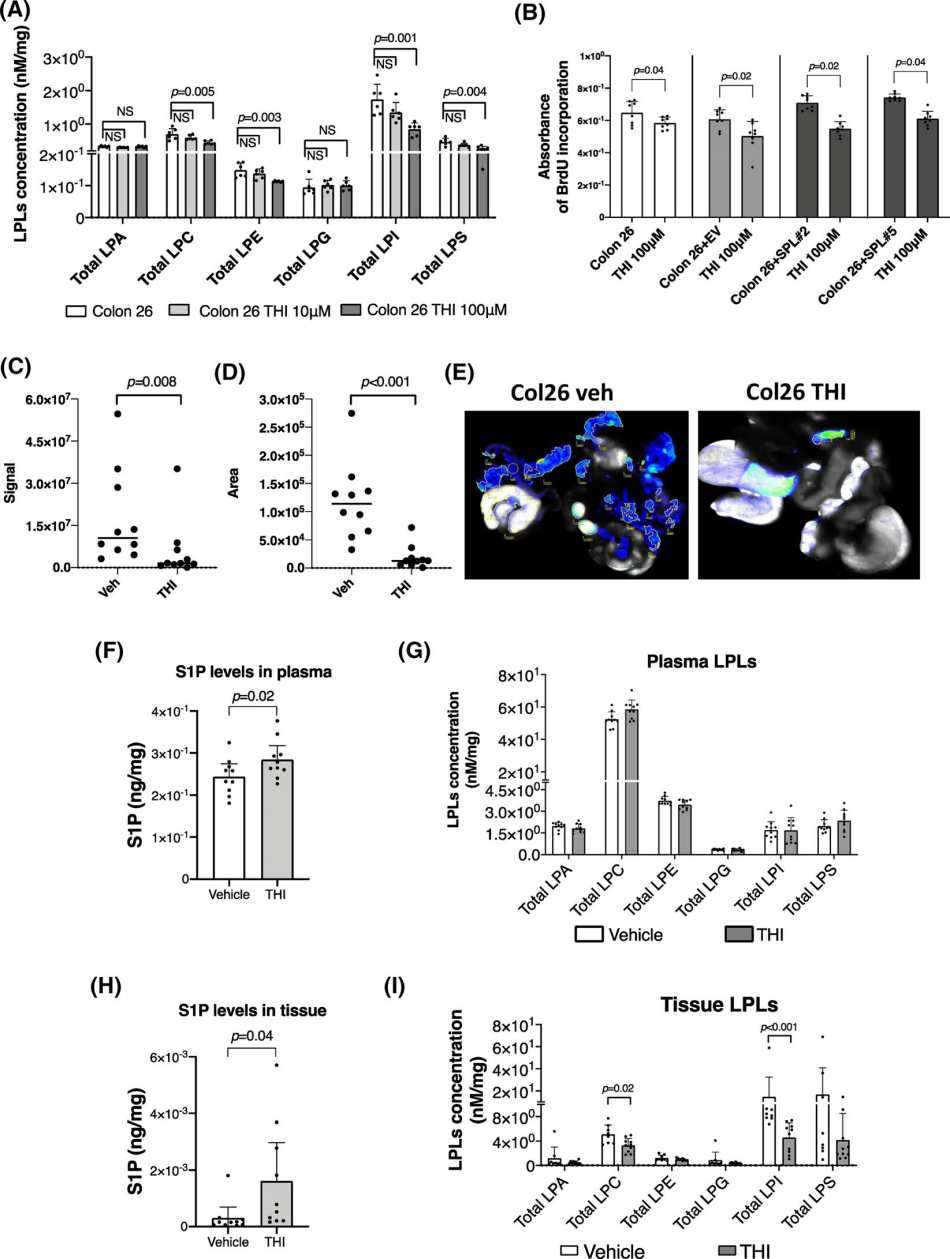
The THI, an inhibitor of the S1P lyase (SPL), worked identical to genetic application both in vitro and in vivo. (A) The dose‐dependent effects of the THI on total levels of the lysophosphatidylcholine (LPC), lysophosphatidylethanolamine (LPE), lysophosphatidylinositol (LPI) in Colon 26 cells. Cells were cultured with 10 μM and 100 μM THI for 72 h and cell lysates including control cell lysates were processed for the measurements of the glyceroLPLs. (B) THI inhibits cell proliferation rates, independently from SPL expression levels. A significant decrease in cell proliferation was observed in cell lines with the addition of the 100 μM THI. Signal (C) and area (D) of the disseminated peritoneal cancer were analyzed in vehicle or THI administered mice by using Odyssey CLx Imaging system. Identical illumination settings (channels: 700/800, resolution: 42 μM, intensities: auto, quality: lowest, analysis: small animal, and focus: 2.0 mm) were used for acquiring all images. (E) Representative scanned images from two groups. Images were acquired and analysed using Image Studio Ver. 4.0 software. (F and H) S1P levels in plasma (F) or disseminated peritoneal cancer tissues (H). S1P levels were significantly increased both in plasma and cancer tissues in THI administered group in comparison to vehicle. (G and I) Total glycero‐LPLs were measured in plasma (G) and disseminated peritoneal cancer tissues (I). In cancer tissues, total LPC and LPI were significantly decreased as an effect of the THI administration. Differences between the two groups were assessed using the unpaired Student's *t*‐test, while differences among more than two groups were assessed using a one‐way ANOVA. EV, empty vector transfected control cell line; SPL, SPL overexpressing cell line

Lastly, we investigated the potential pharmacological implications of the findings of this study, using 2‐acetyl‐4‐(tetrahydroxybutyl) imidazole (THI), a well‐established inhibitor of SPL. We cultured Colon 26 cells with 10 μM or 100 μM THI for 72 h and measured the levels of S1P and glyceroLPLs in the cells. The S1P levels were not affected by THI inhibition (Figure S6G), while the levels of all the glyceroLPLs, except for LPA and LPG, decreased in the cell culture containing the higher concentration of THI (Figure 6A). As compared with that in the cell lines with stably silenced SPL inhibition of SPL by THI might be partial, as it could not overcome the effects of S1P taken up from the culture medium. However, in the presence of 100 μM THI, the cell proliferation ability was inhibited in both the control and SPL‐overexpressing cell lines, suggesting that inhibition of SPL‐mediated pathways, and not of S1P itself, might be involved in the proliferation of the cells in which SPL was pharmacologically inhibited (Figure 6B).

When we administered THI to the mice injected with wild‐type Colon‐26 cells, the levels of S1P in both the plasma and tumour tissues increased (Figure 6F, H). Different from the case in plasma samples (Figure 6G), the total levels of LPC and LPI were decreased in the tumour tissue samples as compared with that in the tissues obtained from vehicle (Figure 6I). Furthermore, oral administration of THI led to successful inhibition of cancer dissemination in this xenograft model (Figure 6C–E, and Figure S6F and S7D).

## 4 DISCUSSION

**FIGURE 7:**
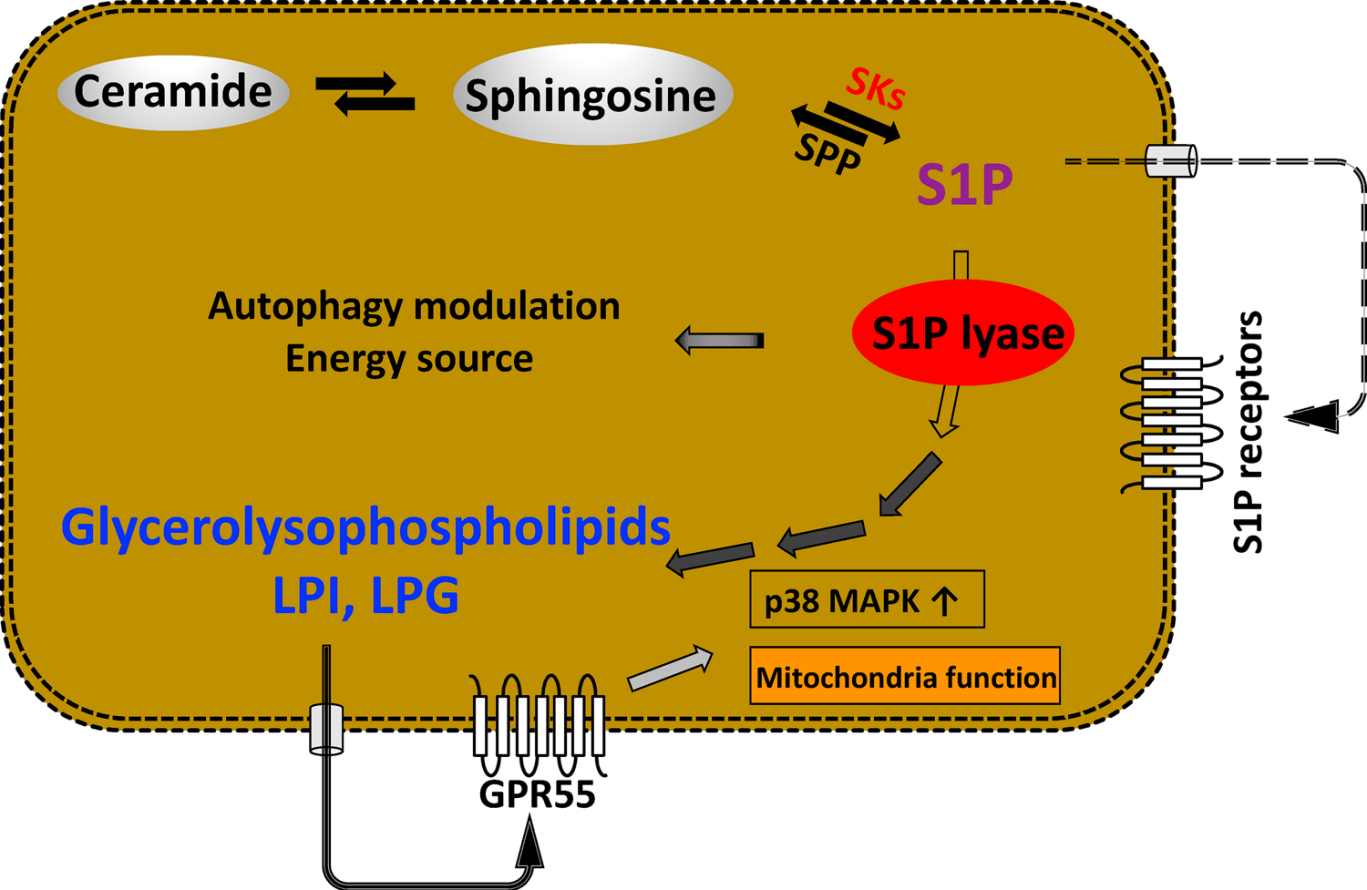
Scheme of the novel metabolic pathway where S1P lyase (SPL) plays a leading role. SPL supports cancer progression as a ligand production through irreversibly degrading S1P and leading to LPI, lysophosphatidylglycerol (LPG) production, strengthening mitochondria function or activating p38 through GPR55, and also inhibiting autophagy which might supply energy required for cancer progression

The present study indicates that SPL, the S1P metabolizing enzyme, has potential roles in the regulation of cancer metabolic pathways, especially by producing LPI and LPG. Findings of clinical studies in HCC and colon cancer patients lend support to this hypothesis and basic studies, including in vitro experiments with Colon 26 cells stably expressing SPL and GPR55, and in vivo experiments using xenograft models have revealed that SPL plays important roles in facilitating cell proliferation via both GPR55‐dependent and GPR55‐independent pathways (Figure 7).

The patient‐based data in this study further endorsed the possibility that SPL might have important roles in generating glyceroLPLs in HCC, because only the mRNA expression levels (tumour/non‐tumour ratio) of SPL, which is also known to function intracellularly,^5^ were correlated with the levels of glyceroLPLs (tumour/non‐tumour ratio), such as LPC and LPI, among the sphingolipid‐related factors (Figure 1, Table S3). As described in *Introduction*, the widely known ceramide‐S1P rheostat might not be a main pathway at least in human HCC and partially in human colon cancer, which we analysed in the present study, based on decreased or unchanged levels of the S1P in tumour tissues in line with increased SPL expression levels.^20^, ^21^ Although the modulations of S1P in cancer might depend on the types of cancer and the backgrounds of the subjects, we believe that SPL might possess more crucial roles at least in some types of cancers. Contrary to the findings of the present study, in one study, the SPL expression level was reported to be lower in colon cancer,^30^ where it appeared to inhibit cancer cell proliferation through induction of apoptosis.

In the present study, however, we used tumour and non‐tumour tissue samples from 148 HCC and 26 colon cancer patients and confirmed our previous findings. Besides its implication in the field of oncology, the SPL‐mediated metabolic pathway, in which sphingolipids are converted to glyceroLPLs, is very important for homeostasis of both sphingo‐ and glyceroLPLs, and the *ALDH3A2* gene involved in this pathway has recently been identified as a cause of the Sjögren–Larsson syndrome,^60^ showing the involvement of this pathway in the development of a variety of diseases.

Contrary to reports from previous studies in colon cancer,^30^, ^61^, ^62^ the present study showed that enhanced expression levels of SPL together with the increased levels of glyceroLPLs, were associated with enhanced cell proliferation abilities of the cells, and that the silencing of SPL was associated with reduced cell proliferation, migration, and invasion abilities, with, in particular, reduction of the levels of LPI and LPG. Treatment of SPL‐silenced cells by exogenous addition of LPI and LPG reversed the decreased cell proliferation ability to the level seen in the control cells, suggesting that glyceroLPLs, especially LPI and LPG, might be responsible for the SPL‐mediated increase in the proliferative ability of cancer cells (Figure 2). Not only final products of the SPL pathway, such as glyceroLPLs, but even intermediate products of this pathway, such as PE and Hexa, reversed the cell proliferation abilities of the SPL‐silenced cell lines (Figure 3). Importantly, addition of these intermediate products led to substantial increases in the levels of LPI and LPG, as well as the levels of other phospholipids which are known to be upstream^35^ of these glyceroLPLs (Figure S1).

GPR55, which is known to be a specific receptor of LPI and partially of LPG^50^, ^51^, ^52^ (Figure 3H), has been reported to be involved in the development of metastasis in colon cancer,^63^ and its down‐regulation was shown to reduce tumour growth in an animal model.^40^ In regard to the mechanisms of involvement of this SPL‐mediated pathway in cancer progression, we propose that both a GPR55‐dependent pathway and GPR55‐independent pathway may be involved. In regard to involvement of the LPI, LPG‐GPR55 axis, silencing or inhibition of the GPR55 receptor inhibited cell proliferation and mitochondrial function, independently of the expression levels of SPL. As cell signaling under GPR55, p38 activation was attenuated by silencing or inhibition of both SPL and the LPI/GPR55 axis (Figure 3). Thus, GPR55 is indispensable in the SPL pathway in cancer cells to induce and support continuous growth of the cells through binding LPI or LPG and furthermore, for activation of the p38 signal cascade, considering the established roles of p38 in the pathogenesis of cancer.^64^ It also known that GPR55 forms heterodimers with cannabinoid receptors as GPCR, and reported to possess important roles in the pathogenesis of cancer especially in breast cancer with unique pharmacological and signaling properties.^65^ Considering these roles together with our finding, GPR55 could be a potential target for anti‐cancer therapies and as a prognostic biomarker.

For the GPR55‐independent pathway, we propose that it is mediated by autophagy. To address the possible mechanistic roles of SPL other than via the LPI, LPG‐GPR55 axis, we checked the condition of autophagy in the cell lines, based on the positive correlations between SPL and autophagy‐related molecules in the clinical study (Table S3), the importance of autophagy in cancer progression,^66^, ^67^ the action of S1P through its receptors as an inhibitor of autophagy via activation of the mTOR pathway,^48^, ^68^ and the direct role of SPL in neuronal autophagy.^69^ Autophagy has dual roles in cancer, with both tumour‐promoting^70^, ^71^, ^72^ and tumour‐suppressing properties^73^, ^74^, ^75^; mostly, it is strongly up‐regulated in cancer cells to enable them to survive under tough conditions,^76^ by capturing unnecessary or dysfunctional proteins and organelles within the cells for degradation and recycling for further use with the participation of autophagy‐related genes.^77^ In addition, phosphatidylethanolamine, which is one of the intermediate products of S1P degradation, is known to act as an anchor for LC3 and to be involved in cargo recruitment and formation of autophagosomes. Contrary to our expectation of up‐regulation of autophagy in the cancer cells, however, we observed reduction of LC3‐II in the SPL‐overexpressing cell lines, together with enhanced phosphorylated mTOR and p70S6K, which are known to contribute to the regulation of autophagy (Figure 5A), suggesting suppression of autophagy in the SPL‐overexpressing cell lines. In the metabolomics analyses, we observed no modulation of the amino acid levels by SPL, even under the glucose‐absent condition, suggesting that the decreased autophagy induced by SPL overexpression might not inhibit cell proliferation in the aspects of the complement of depleted nutrition. Instead, we proposed that the decreased autophagy induced by SPL overexpression might prevent the degradation of EGFR. EGFR is commonly highly expressed in many cancers^78^ and EGFR itself or its downstream signaling pathways determine whether autophagy will be up‐regulated or down‐regulated in cancers.^79^, ^80^ EGFR‐mediated pathways, such as the RAS (rat sarcoma virus, a class of protein called small GTPase) pathway, STAT3 pathway and above‐ mentioned mTOR pathway are involved in the regulation of autophagy, which is complex and still remains to be clearly elucidated.

In metabolomics analyses, we observed significant increases in the levels of metabolites serving as energy sources (e.g., glucose 6‐phosphate) and involved in cell membrane stabilization (e.g., taurine) and protein traffic (e.g., mannose 6‐phosphate) in the SPL‐overexpressing cell lines. Concordantly, we observed increased mitochondrial membrane potential following the addition of PE in the SPL‐silenced cell lines, although the direct involvement of increased mitochondrial functions in cancer progression remained unsolved in the present study.

Once again, this study raised the possibility that the ceramide‐S1P rheostat may, not be fully operational in all cancers, especially in cancers in which not only SPL, but also other enzymes responsible for producing LPI and/or LPG from sphingolipids via PE were enhanced, based mainly on our HCC‐related study.^20^ Therefore, we think that the levels of PE and LPE, which is produced form PE, were not higher in cancers. This metabolic pathway is also thought to exist in colon cancer,^21^ although the S1P release system is also elevated in most colon cancers and the S1P receptors are also increased in both HCC and colon cancers; according to another group, overexpression of SPL led to colon cancer cell apoptosis.^30^ Actually, in esophageal cancer, we observed only enhanced expression levels of S1P_2_, S1P_3_ and S1P_5,_ with no significant changes in the expression levels of the SKs or SPL (Figure S2A). According to the Cancer Genome Atlas Program, cancers of the ovary, bronchus and lung also show increased expression levels of SPL.^81^ These results suggest the possible contribution of the SPL‐mediated metabolic pathway, presented in this study, to the pathogenesis of cancer might depend on the type of cancer. SPL is responsible for irreversible degradation of S1P, as described in the *Introduction* section, therefore, SPL may regulate not only the intracellular levels of S1P, but also the amount of S1P available for extracellular export, by influencing autocrine or paracrine signaling through extracellular S1P receptors, especially in colon cancer, in which the S1P release system is up‐regulated.^21^ Moreover, the importance of the sphingolipids in chronic inflammation of the digestive system has accumulated attentions and these recent advances could promote the therapeutic applications, targeting SKs, S1P and S1PRs.^82^, ^83^, ^84^ Therefore, both the SPL‐mediated pathway and S1P‐S1P receptors might be useful therapeutic targets for the treatment cancer and the importance of these targets might depend on the cancer species. Further studies are necessary to address these points.

As the main tool for preclinical testing of drugs in cancer research, researchers often use nude mice with an inhibited immune system. S1P and glyceroLPLs have been demonstrated to be involved in immune response to tumour.^49^, ^85^ Therefore, in this study, to mimic the clinical situation in the human organism (the immune system is present), we used normal experimental mice. Regarding other limitations of the present study, the modulations of autophagy by SPL have not been fully elucidated and, since autophagy is involved in various diseases, further careful studies on the associations between SPL and autophagy are necessary. As shown in Figure S2 and described above, the modulations of SPL were not observed in esophageal cancer, suggesting that the roles of SPL and SPL‐mediated pathway might depend on cancer types or conditions. Further studies on this point are also needed to introduce the findings of the present study into clinical application.

Lastly, to confirm the possible pharmacological implication of the present findings, we demonstrated different rates of tumour progression using not only cell lines with modulated SPL expressions, but also an SPL inhibitor. To the best of our knowledge, this report is the first to show that the small‐molecule food colorant 2‐acetyl‐4‐tetrahydroxybutylimidazole (THI) administered orally in drinking water could inhibit cancer progression by inhibiting SPL in vivo (Figure 6C–E, Figure S7A). The usefulness of THI in the early stage after acute myocardial infarction,^59^, ^86^ in reducing the infarct size and enhancing hemodynamic recovery,^87^ and in inhibiting lymphocyte egress from the infarct area,^88^ has been reported previously. Further studies are necessary to finalize the effect of the THI in cancer progression because THI is also suggested to be a direct inhibitor of pyridoxal kinase and might inhibit SPL directly or inhibit the cofactors required to catalyse S1P irreversibly through pyridoxal kinase inhibition.^89^ Therefore, there also remains the possibility that several other enzymes linked to pyridoxal kinase inhibition such as vitamin B6‐dependent enzymes could be affected by THI administration in these experiments. The direct effect of THI against cancer progression and a similar effect of THI as that obtained by silencing of SPL by the Crispr/Cas9 system is one of our additional findings in this field.

## 5 CONCLUSION

Taken together, these results suggest that, in HCC and colon cancer, SPL supports cancer progression through irreversibly degrading S1P and increasing glyceroLPL production, and also as an energy supplier. In this study, we showed the association between SPL and cancer and elucidated a novel metabolic pathway that appears to exist in several kinds of cancer cells (Figure 7).

## CONFLICT OF INTEREST

The authors declare no conflict of interest.

## Supporting information

Supporting Information
