# Supplementary material for: Sphingosine 1‐phosphate lyase facilitates cancer progression through converting sphingolipids to glycerophospholipids
Source: Clin Transl Med. 2022 Sep 20;12(9):e1056. doi: 10.1002/ctm2.1056 (PMC9488530; doi:10.1002/ctm2.1056)
Supplement: Supplementary file 1 — Supporting Information [file CTM2-12-e1056-s001.docx]

**Supplementary Table 1**

**A.** Correlation between sphingolipid related factors in HCC.

|  |  | **SK1 T/Nt** | **SK2 T/Nt** | **S1P1 T/Nt** | **S1P2 T/Nt** | **S1P3 T/Nt** | **SPL T/Nt** | **SPP1 T/Nt** | **SPNS2 T/Nt** | **FALDH T/Nt** |
| --- | --- | --- | --- | --- | --- | --- | --- | --- | --- | --- |
| **SK1 T/Nt** | rho |  |  |  |  |  |  |  |  |  |
|  | *p* value |  |  |  |  |  |  |  |  |  |
| **SK2 T/Nt** | rho | 0.1319 |  |  |  |  |  |  |  |  |
|  | *p* value | 0.11 |  |  |  |  |  |  |  |  |
| **S1P1 T/Nt** | rho | 0.1489 | 0.4037 |  |  |  |  |  |  |  |
|  | *p* value | 0.0708 | <0.0001 |  |  |  |  |  |  |  |
| **S1P2 T/Nt** | rho | 0.4445 | 0.3347 | 0.2851 |  |  |  |  |  |  |
|  | *p* value | <0.0001 | <0.0001 | 0.0004 |  |  |  |  |  |  |
| **S1P3 T/Nt** | rho | 0.4085 | 0.1880 | 0.4252 | 0.4449 |  |  |  |  |  |
|  | *p* value | <0.0001 | 0.0221 | <0.0001 | <0.0001 |  |  |  |  |  |
| **SPL T/Nt** | rho | 0.1485 | 0.5465 | 0.2397 | 0.3719 | 0.1739 |  |  |  |  |
|  | *p* value | 0.07 | <0.0001 | 0.0033 | <0.0001 | 0.0345 |  |  |  |  |
| **SPP1 T/Nt** | rho | 0.1652 | 0.4248 | 0.3355 | 0.3396 | 0.2895 | 0.5470 |  |  |  |
|  | *p* value | 0.044 | <0.0001 | <0.0001 | <0.0001 | 0.0004 | <0.0001 |  |  |  |
| **SPNS2 T/Nt** | rho | 0.2610 | 0.2541 | 0.2558 | 0.4005 | 0.4002 | 0.2723 | 0.3692 |  |  |
|  | *p* value | 0.0014 | 0.0018 | 0.0017 | <0.0001 | <0.0001 | 0.0008 | <0.0001 |  |  |
| **FALDH T/Nt** | rho | -0.04385 | 0.4012 | 0.3132 | 0.2356 | 0.09310 | 0.5371 | 0.4118 | 0.3146 |  |
|  | *p* value | 0.5967 | <0.0001 | 0.0001 | 0.0039 | 0.2604 | <0.0001 | <0.0001 | <0.0001 |  |
| **CTP T/Nt** | rho | -0.1271 | 0.5248 | 0.3227 | 0.2779 | 0.03296 | 0.5715 | 0.4031 | 0.2008 | 0.7008 |
|  | *p* value | 0.1236 | <0.0001 | <0.001 | 0.0006 | 0.6909 | <0.0001 | <0.0001 | 0.0144 | <0.0001 |

**B.** Correlation between sphingolipid related factors in Colon cancer.

**C.** Correlations of SPL between sphingolipid related factors in the pan-cancer study (TCGA).

**Supplementary Table** **2.** Sphingolipid related factors’ correlation with various markers

**A.** In HCC

**B.** In colorectal cancer

**C.** Correlations of SPL with various markers in the pan-cancer analysis (from TCGA)

**Supplementary Table 3**. Sphingolipid related factors’ correlation with tissue PL and LPLs

**Supplementary Fig. 1. The metabolic pathway scheme of the sphingolipid to glycerolysophospholipid (glyceroLPLs) pathway.**

**Supplementary Fig. 1.** SPL plays a crucial role to convert sphingolipids to glyceroLPLs through irreversibly degrading S1P which is produced from sphingosine by SKs. Hexadecenal and phosphoethanolamine are direct products from SPL action, which are inevitable in further production of the phospholipids and glyceroLPLs as final products of this pathway.

**Supplementary Fig. 2. The mRNA expression levels of sphingolipid related factors in tumor and non-tumor tissues of esophageal cancer and the calibration curves used for calculation of the concentrations of S1P, PLs, and glycero-LPLs.**

**Supplementary Fig. 2. A.** A total of N=33 patients’ tumor and non-tumor paired samples were used for the measurements of the mRNA expression levels. S1P_2_, S1P_3_, and S1P_5_ receptors expression levels were significantly increased in tumor tissues in comparison to its adjacent non-tumor tissues. Differences between the two groups were assessed using the Wilcoxon signed-rank test .

**B.** The results of the calibration curves of the S1P. The lower limit of quantification was 0.01ng/mL for S1P. Linearity was confirmed over the entire range of the tested concentrations of standard solutions (0–100 ng/mL).

**C, D.** The results of the calibration curves of the phospholipids (PC, PI, PG, PE) and glyceroLPLs (LPC, LPS, LPE, LPA). The lower limit of quantification was 0.001 nM for all standard solutions. Linearity was confirmed over the entire range of the tested concentrations of standard solutions (0–100 nM).

**Supplementary Fig. 3. Modulation of the SPL expression level influences levels of the glyceroLPL levels.**

**Supplementary Fig. 3. A.** Representative cell colonies transfected with the targeting vector. Cell colonies that were transfected with empty vector (EV) were negative for RFP, and SPL-silenced cell colonies were positive or RFP in selection culture medium with puromycin. **B.** Genotyping analysis of the selected cell colonies transfected with control or targeting vector. Primers for confirmation designed to get around 1000-bp sized PCR products (white arrow indicates 1000 bp). #2 and #6 cell lines were chosen for further experiments. **C.** Total glycero-LPL levels in the cell lines (N=6) in the presence of glucose. **D-H.** Each species of the LPC, LPI, LPS, LPE, and LPA were measured in the SPL-overexpressing cell lines (N=6) in the absence of glucose. **I.** Total glycero-LPL levels in the SPL-silenced cell lines (N=6) in the presence of glucose. Total LPI and LPG levels were significantly decreased in the SPL-inhibited cells as compared to the control cells. **J-M.** Each species of LPI, and LPG was also measured in SPL-inhibited cell lines in the absence or presence of glucose (N=6). **N.** Representative transwell migration and invasion assay images (200× magnification) from SPL overexpressing or silenced cell lines in comparison to control cell lines. Cell migration was increased in Colon 26 cell lines with the overexpression of SPL, while no changes were observed in invasion ability. SPL silencing reduced both cell migration and invasion in comparison to control cell lines. **O.** SPL mRNA expression levels in individual single-cell colonies. Low expressing cell lines circled in green. Among these single-cell colonies moderate (Colon26+SPL#2), or high (Colon26+SPL #5) SPL expressing cell lines (red circled) were used for further experiments. **P.** Total glycero-LPLs levels were measured in cell lines with low levels (Colon26+SPL#1, Colon26+SPL#6), and moderate levels (Colon26+SPL#2), and high levels (Colon26+SPL#5) of the SPL by comparing with control cell lines (Colon 26 and Colon26+EV). Total glycero-LPLs levels were significantly increased in cells where SPL expressed in moderate or high levels in comparison to control or low expressing cell lines (N=6 from each cell line). (*) indicates differences with Colon 26 , (#) indicates differences with EV, (&) indicates differences with #1 SPL overexpressing cell line, ($) indicates differences with #6 SPL overexpressing cell line.

(*), (#), (&), ($): p<0.05; (**), (##), (&&), ($$): p<0.01; (***), (###), (&&&), ($$$): p<0.001;

Differences among more than two groups were statistically analyzed by one-way ANOVA.

EV: empty vector transfected control cell line

HR: homologous recombination vector only transfected control cell line

SPL: SPL overexpressing cell line

SPL HR: homologous recombination vector transfected SPL overexpressing cell line

Cr/Cas SPL: SPL knock out cell lines by using CRIPR/Cas9 system

**Supplementary Fig. 4. In-vitro experiments in human HCC and colon cancer cell lines.**

**Supplementary Fig. 4. A, C, E, G.** mRNA expression levels of SPL (N=3). In HuH7 and LoVo cells, the SPL levels were overexpressed (A), in PLC/PRF/5 and HCT116 cells, SPL levels were inhibited, where SPL was transiently silenced by using siSPL with siNC (C). **B, D, F, H.** The total glycero-LPL levels were measured in SPL overexpressed HuH7 and LoVo cell lines or SPL-silenced PLC/PRF/5 and HCT116 cell lines (N=6). Total LPG and LPI levels were increased in the HuH7 cell lines (B) and total LPG was decreased in the PLC/PRF/5 cell lines (D). In LoVo cell lines total LPG, LPI, and LPS were increased, while total LPC, LPG, and LPS were decreased in HCT116 cell lines.

Differences between the two groups were statistically analyzed by the unpaired Student's t-test.

EV: empty vector transfected control cell line

SPL: SPL overexpressing cell line

siNC: Negative control siRNA transfected cell line

siSPL: SPL silencing siRNA transfected cell line

**Supplementary Fig. 5. Addition of PE and Hexa, the intermediate products, increases the PL and glycero-LPL levels in SPL-inhibited cell lines.**

**Supplementary Fig. 5. A.** GPR55 expression level in Colon 26 cell lines. RT-PCR products (420 bp) loaded in 2% agarose gel, with mouse spleen tissue samples used as positive control, and mouse liver tissue samples used as negative control. **B.** Cell proliferation assay in SPL-overexpressing cell lines (N=8) with the addition of CID, a GPR55 inhibitor, at the concentration of 2.5 µM, 5.0 µM, 10.0 µM respectively. **C-E.** PE, PS and PC levels in the presence of the PE, **F-H** in the presence of the Hexa. Levels of the PE, PS, and PC were measured by LC-MS/MS and (&) indicates the difference between the control and SPL-inhibited cell lines, (*) indicates the difference between the control and the SPL-inhibited cell lines added with PE or Hexa, (#) indicates the difference between the SPL-inhibited cell lines added with PE or Hexa and SPL- inhibited cell lines without the addition of PE or Hexa. **I-K.** The same cells (N=6) were used for the measurements of the total LPC, LPE, and LPS. (*) indicates the difference between the control and the SPL-inhibited cell lines added with PE or Hexa , (#) indicates the difference between the SPL-inhibited cell lines added with PE or Hexa and SPL- inhibited cell lines without the addition of PE or Hexa. **L.** The representative fluorescence images of the mitochondrial membrane potential in the SPL-inhibited cell lines and those added with PE. The addition of PE increased mitochondrial membrane potential, which is confirmed by decreased monomer formation. **M.** Mitochondrial membrane potential was measured in the GPR55-inhibited cell lines with or without SPL overexpression by adding LPI 18:1 (1 µM), LPG 18:1 (1 µM), PE (5 µM), and Hexa (10 µM). There were no changes with the addition of any of the compounds in a glucose-absent condition (N=8 from each cell line). (*) indicates differences with HR control cell line, (#) indicates differences with SPL overexpressing HR control cell line. **N.** Cell proliferation assays were performed in Colon26+SPL#2 cell lines with or without the addition of the Rapamycin (100nM) in a glucose-present or a glucose-absent condition (N=8 from each cell line). **O.** The same cell lines and experimental conditions were used for the measurement of the mitochondrial membrane potential (N=8 from each cell line). When treated with Rapamycin, cell proliferation and mitochondrial membrane potential were not enhanced in the SPL overexpressing cell lines. **P.** WB analysis of the total and phosphorylated p38 MAPK in Col 26 and Col 26+SPL#2 cell lines. The phosphorylation of the p38 MAPK was increased in the SPL overexpressing cell lines in comparison to the control Col 26 cell lines when cultured 24 hrs in serum- and glucose-free conditions. **Q.** Quantification of WB by densitometry using the NIH ImageJ application. WB bands from 6 repeats of the same set of samples were used, and the ratios of the p-p38 intensity to the total p38 intensity were quantified.

Differences between the two groups were statistically analyzed by the unpaired Student's t-test, while differences among more than two groups were statistically analyzed by one-way ANOVA.

EV: empty vector transfected control cell line

HR: homologous recombination vector only transfected control cell line

+SPL: SPL overexpressing cell line

Cr/SPL: SPL knock out cell lines by using CRIPR/Cas9 system

Cr/SPL+PE or Hexa: phosphoethanolamine (PE) or hexadecenal (Hexa) added in culture medium of the SPL knock out cell lines by using CRIPR/Cas9 system

Cr/GPR55: GPR55 knock out cell lines by using CRIPR/Cas9 system

Cr/GPR55 LPI, or LPG, or PE, or He: GPR55 knock out cell lines by using CRIPR/Cas9 system with addition of the LPI, or LPG, or PE, or He.

SPL Cr/Cas GPR55: GPR55 knock out cell lines by using CRIPR/Cas9 system in SPL overexpressing cell line

SPL Cr/Cas GPR55 + LPI, or LPG, or PE, or He: GPR55 knock out cell lines by using CRIPR/Cas9 system in SPL overexpressing cell line with addition of the LPI, or LPG, or PE, or He.

**Supplementary Fig. 6. In-vivo experiments to evaluate the effects of modulated SPL expression on peritoneal cancer dissemination.**

**Supplementary Fig. 6. A.** Glucose uptake rates in the cancer cell lines (N=8) used for peritoneal injection were measured to evaluate the effects on cancer progression. There were no differences in the glucose uptake rates among the cell lines used for the in-vivo experiments (NS=not significant). **B.** The in-vivo experiment schedule. We injected (2×10^6^ cells in 100 µl PBS) cells from 8 different cell lines (EV, SPL, SPL+HR, SPL+Cr/GPR55, HR, Cr/SPL, Colon26, Colon26+THI) intraperitoneally on day 0, IRDye 2-DG was injected into the tail vein of the mice on day 8, and on day 10, we collected samples for further analysis. For each cell line, N=10 mice were used. **C-F.** All scanned images for each group (N=10) were acquired and analyzed using the Image Studio Ver. 4.0 software. **G.** S1P levels in Colon 26 cell lines cultured under THI (10 µM and 100 µM)-added culture conditions for 72 hours. **H.** WB analysis of the proteins involved in autophagy or mitochondrial functions in disseminated peritoneal cancer tissue samples described in Fig.4 and Fig. 6C-E.

EV: empty vector transfected control cell line

HR: homologous recombination vector only transfected control cell line

SPL: SPL overexpressing cell line

Cr/SPL: SPL knock out cell lines by using CRIPR/Cas9 system

Cr/GPR55: GPR55 knock out cell lines by using CRIPR/Cas9 system

SPL HR: homologous recombination vector transfected SPL overexpressing cell line

SPL Cr/Cas GPR55: GPR55 knock out cell lines by using CRIPR/Cas9 system in SPL overexpressing cell line

**Supplementary Fig. 7. Representative of histopathology of the disseminated peritoneal cancer tissues.**

**Supplementary Fig. 7. A-C.** Representative of the histopathology (HE) images, and an original photos of the peritoneal organs , mainly intestine, spleen, pancreas (left side), and liver (right side), when injected following set of the cells. Control Colon26 cells vs SPL overexpressing cells (A), SPL overexpressing cells vs SPL overexpressing but GPR55 inhibited cells (B), and control Colon 26 cell vs SPL inhibited cells (C). Peritoneal cancer dissemination was dependent on both the SPL expression levels and GPR55 expression levels. Peritoneal disseminated cancer tissues developed by cell injection pointed by red arrowhead. **D.** The upper section describes HE stain, and an original photos of the peritoneal organ, mainly intestine, spleen, pancreas (left side), and liver (right side), of the vehicle group where Colon26 cells were injected. The lower section displays the peritoneal organ of the THI administered group with an injection of the same Colon26 cells peritoneally. Disseminated cancer tissues (pale purple color) which are pointed by red arrowhead enlarged in red rectangles. THI administration decreased the formation of peritoneal cancer by inhibiting SPL activity.

EV: empty vector transfected control cell line

HR: homologous recombination vector only transfected control cell line

SPL: SPL overexpressing cell line

Cr/SPL: SPL knock out cell lines by using CRIPR/Cas9 system

SPL HR: homologous recombination vector transfected SPL overexpressing cell line

SPL Cr/Cas GPR55: GPR55 knock out cell lines by using CRIPR/Cas9 system in SPL overexpressing cell line
